# Enterovirus evolution reveals the mechanism of an RNA-targeted antiviral and determinants of viral replication

**DOI:** 10.1126/sciadv.adg3060

**Published:** 2024-02-16

**Authors:** Jesse Davila-Calderon, Mei-Ling Li, Srinivasa R. Penumutchu, Christina Haddad, Linzy Malcolm, Josephine King, Amanda E. Hargrove, Gary Brewer, Blanton S. Tolbert

**Affiliations:** ^1^ Department of Chemistry, Case Western Reserve University, Cleveland, OH, USA.; ^2^ Department of Biochemistry and Molecular Biology, Rutgers Robert Wood Johnson Medical School, Piscataway, NJ, USA.; ^3^ Department of Chemistry, Duke University, Durham, NC, USA.; ^4^ Department of Biochemistry and Biophysics, University of Pennsylvania, Philadelphia, PA, USA.; ^5^ Howard Hughes Medical Institute, Chevy Chase, MD, USA.

## Abstract

Selective pressures on viruses provide opportunities to establish target site specificity and mechanisms of antivirals. Enterovirus (EV)-A71 with resistant mutations in the stem loop (SL) II internal ribosome entry site (IRES) (SLII^resist^) were selected at low doses of the antiviral dimethylamiloride (DMA)-135. The EV-A71 mutants were resistant to DMA-135 at concentrations that inhibit replication of wild-type virus. EV-A71 IRES structures harboring resistant mutations induced efficient expression of Luciferase messenger RNA in the presence of noncytotoxic doses of DMA-135. Nuclear magnetic resonance indicates that the mutations change the structure of SLII at the binding site of DMA-135 and at the surface recognized by the host protein AU-rich element/poly(U)-binding/degradation factor 1 (AUF1). Biophysical studies of complexes formed between AUF1, DMA-135, and either SLII or SLII^resist^ show that DMA-135 stabilizes a ternary complex with AUF1-SLII but not AUF1-SLII^resist^. This work demonstrates how viral evolution elucidates the (DMA-135)–RNA binding site specificity in cells and provides insights into the viral pathways inhibited by the antiviral.

## Teaser

Enterovirus evolution reveals the mechanism of an RNA-targeted small molecule and determinants of virus replication.

## INTRODUCTION

Non-polio human enteroviruses (EVs) are positive strand RNA pathogens that pose a serious threat to global economies and health care infrastructures; nevertheless, there are no commercially available antivirals to thwart community outbreaks. EVs infect millions of people around the world and thousands in the United States annually. Although infections typically manifest with mild and self-limiting illness, prolonged infections in the immunocompromised can lead to severe neurological disorders, cardiopulmonary failure, and death (*1*–*7*). Thus, EV infections have the potential to develop into severe health outcomes given that disease pathogenesis impairs multiple organ systems (*8*).

Transmission of EV-A71, an etiological agent of the hand, foot, and mouth disease, has become endemic to the Asia-Pacific region with major outbreaks every 3 to 4 years such as a 2018 epidemic in Vietnam where more than 53,000 hospitalizations and 6 deaths were reported (*9*). The National Institute of Allergy and Infectious Diseases recognized EV-A71 and related EV-D68 as group II reemerging pathogens. As of the time of writing, treatment of EV-A71/D68 infections remains largely supportive because there are no Food and Drug Administration–approved vaccines or therapeutics, emphasizing the need to develop a comprehensive understanding of the molecular mechanisms involved in host-virus interactions.

EV-A71 is a nonenveloped, single-stranded, positive-sense RNA virus found in species A of the *Enterovirus* genus within the *Picornaviridae* family. Its 7500-nucleotide genome serves as template for viral translation and replication by partitioning these functions via changes to the host-virus interactions that regulate the cellular stages of viral replication (*10*). The viral genome encodes a single 250-kDa polyprotein using a long open reading frame (ORF) that is flanked by highly structured untranslated regions (UTRs). Given this limited coding capacity, EV-A71 coordinates complex molecular events to usurp host proteins to drive translation of the viral polyprotein (*11*). Notably, EV-A71 uses a type-I internal ribosome entry site (IRES) within its 5′UTR to initiate translation in a cap-independent mechanism assisted by cellular RNA-binding proteins (RBPs), collectively known as ITAFs (IRES trans-acting factors) (*12*, *13*). IRES-mediated translation commences immediately following infection where ITAFs modulate the efficiency by which the ribosome loads internally onto the 5′UTR (*14*). Thus, IRES-ITAF interactions are essential determinants of the earliest fates of EV-A71 replication, making them attractive targets for therapeutic intervention (*11*, *15*).

The IRES of EV-A71 is predicted to fold into five major stem loops (SLs II to VI) in addition to the 5′-end cloverleaf structure required for virus replication. SLII is the only domain whose structure is solved at high resolution, and several of its binding partners have been functionally validated (*15*–*21*). The structure of EV-A71 SLII consists of a phylogenetically conserved 5-nt bulge and 7-nt apical loop (*16*, *21*). Mutations or deletions to the bulge sequence impair viral replication by attenuating IRES-dependent translation (*16*, *21*). SLII binds several cellular RBPs and viral-derived small RNA 1 (vsRNA1) (*16*–*21*). Of the RBPs, heterogenous ribonucleoprotein (hnRNP) A1 and AU-rich element/poly(U) binding/degradation factor 1 (AUF1) are essential ITAFs that exert regulation by competitively binding SLII to differentially modulate viral translation levels (*19*, *20*). HnRNP A1 stimulates IRES-dependent translation, whereas AUF1 antagonizes binding of hnRNP A1 to down-regulate polyprotein synthesis. In a recent study, we leveraged the functional significance of the SLII IRES domain to screen a library of small molecules and identified the dimethylamiloride-135 (DMA-135) as a potent inhibitor of EV-A71 replication (*15*). We demonstrated that DMA-135 attenuates IRES-dependent translation by binding the bulge loop to allosterically stabilize a (DMA-135)–SLII-AUF1 ternary complex that, we posited, disrupts the homeostatic balance of the SLII-host regulatory axis (*15*). The cellular mechanism by which DMA-135 exerts its antiviral properties remained unknown, however.

Herein, we harnessed the power of viral evolution to establish the cellular mechanism of DMA-135 and to reveal additional insights into EV biology. By treating EV-A71–infected cells with an inhibitory dosage of DMA-135, we were able to select for viruses that grow to high titers after 10 rounds of serial passage. Sequencing the 5′UTR of a plaque-purified virus revealed that the resistance mutations mapped to sites adjacent to the bulge motif in the SLII domain, the binding site of DMA-135 (*15*). Specifically, residues C132 and A133 were changed to G132 and C133 in a noncompensatory manner in the resistant SLII RNA (SLII^resist^). Of significance, genetically engineered EV-A71 harboring only the C132G and A133C mutations were shown to replicate with uncompromised efficiencies even when exposed to DMA-135 concentrations that completely inhibit the wild-type virus. Dual Luciferase reporter constructs that contain SLII^resist^ retained normal IRES-dependent Luciferase activities with and without inhibitory levels of DMA-135.

To better understand the functional specificity of DMA-135, we carried out a comparative biophysical analysis of wild-type SLII and the resistant mutant. Nuclear magnetic resonance (NMR) studies indicate that the resistant mutations change the structure of SLII proximal to the bulge loop while preserving the overall folding arrangement of the upper helix, including the 7-nt apical loop. Calorimetric titrations determined that hnRNP A1 binds SLII^resist^ with comparable thermodynamic properties to the wild-type SL. By contrast, AUF1 does not bind the resistant mutant detectably by calorimetry or by a biochemical pull-down assay. As anticipated, DMA-135 does not promote the formation of an allosteric ternary complex with AUF1 and the SLII^resist^ construct. We also demonstrated that DMA-135 functions specifically on the AUF1-SLII complex and not on other known cellular AUF1-dependent RNAs. Collectively, these results support that the mechanism of action of DMA-135 is to tip the SLII-host regulatory axis toward substantially lower levels of IRES-dependent translation, and the virus can compensate by evolving mutations that restore homeostasis.

We also show that DMA-135 can inhibit replication of the related EV-D68 Fermon variant (*22*) albeit less efficiently than EV-A71. NMR comparison of the SLII structures of EV-A71 and EV-D68 reveal that the RNAs adopt similar global folds but different structures within the vicinity of the DMA-135 binding epitope. Notably, EV-D68 consists of an internal loop in place of the 5-nt bulge found in EV-A71. Not only is the internal loop topologically different than the bulge but its sequence composition also varies from the high-affinity AUF1-binding motif found in SLII from EV-A71. Our data reveal that DMA-135 binds specifically to SLII from EV-D68, and it modestly increases the affinity for AUF1, similar to its mechanism observed for EV-A71. Together, the work here defines the antiviral mechanism of action of DMA-135; it demonstrates that functional specificity can be modulated through natural and drug-dependent viral evolution; and it shows how small molecules can reveal additional insights about host-virus interfaces that regulate early stages of EV replication. We expect that such studies will prove beneficial for efforts to target viral RNA structures or complexes with small molecules and to develop chemical biology reagents that can inform on the cellular stages of viral replication.

## RESULTS

### Selection of DMA-135 resistant EV-A71 virus harboring mutations in the SLII IRES domain

As previously reported, DMA-135 functions as an inhibitor of EV-A71 translation and replication (*15*). We further demonstrated that the small molecule targets the bulge motif in the SLII IRES domain to induce a conformational change to the RNA structure within and adjacent to the bulge loop. Notably, the changes disrupt the A133-U163 base pair and abrogate sequential stacking of the AAU sequence motif of the bulge. The exposed AAU motif forms part of the high-affinity binding site for AUF1 (*15*).

**Fig. 1.**
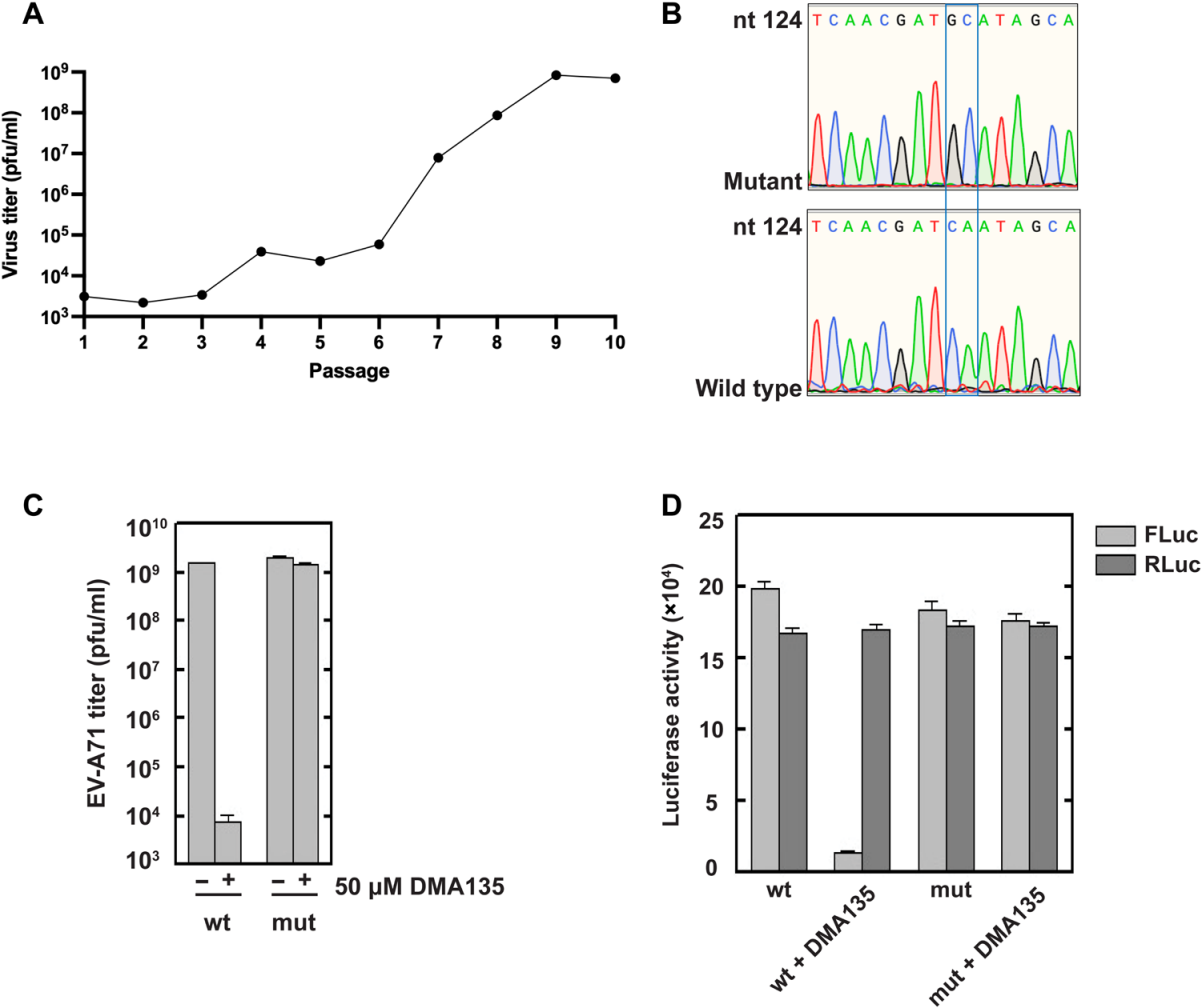
Pressure-induced viral evolution selects for mutant viruses resistant to DMA-135. (**A**) Identification of a (DMA-135)–resistant virus. SF268 cells were infected with undiluted EV-A71 virus stock in the presence of 50 μM DMA-135 for 24 hours. Culture medium was serially passaged in the presence of drug for nine additional rounds. Each passage of medium was tittered by plaque assay with Vero cells. Titer numbers were plotted versus passage number. One plaque from passage 9 was purified for further analyses. (**B**) Sequence comparison of wild-type and mutant EV-A71 genome selected after serial passage 9. The region shown corresponds to the 5′-half of SLII that includes the bulge loop. The sequence comparison shows that the mutant virus incorporated two nucleotide substitutions (C132G and A133C) in SLII. (**C**) Effect of DMA-135 on an engineered mutant EV-A71 virus harboring the C132G and A133C SLII mutations exclusively (SLII^resist^). Wild-type virus or SLII^resist^ virus (mut) were used to infect SF268 cells at a multiplicity of infection = 1, with or without 50 μM DMA-135. After 24 hours, virus titers were determined. Titers were plotted versus the noted conditions (*N* = 3). (**D**) Effect of DMA-135 on bicistronic, luciferase reporter RNAs harboring either the wild-type or SLII^resist^ mutations. The respective IRES drives cap-independent translation of the firefly luciferase ORF. Renilla luciferase serves as the cap-dependent translation control for both RNAs. Luciferase activity is in arbitrary units.

To evaluate the in vivo mechanism of action of DMA-135, we grew (DMA-135)–resistant viruses by repeated culturing of the wild-type virus for 10 passages at a fixed concentration (50 μM) of the inhibitor. The virus titers for each passage were determined by plaque assay (Fig. 1A). Initial resistance to DMA-135 was observed after passage 4 (3.9 × 10^4^ pfu/ml). This resistance improved over passages 5 to 9 (8.4 × 10^8^ pfu/ml). The virus titers obtained in passage 9 were comparable to those obtained when using the wild-type EV-A71 strain in the absence of the inhibitor (Fig. 1A), indicating the generation of a (DMA-135)–resistant, mutant EV-A71.

**Fig. 2.**
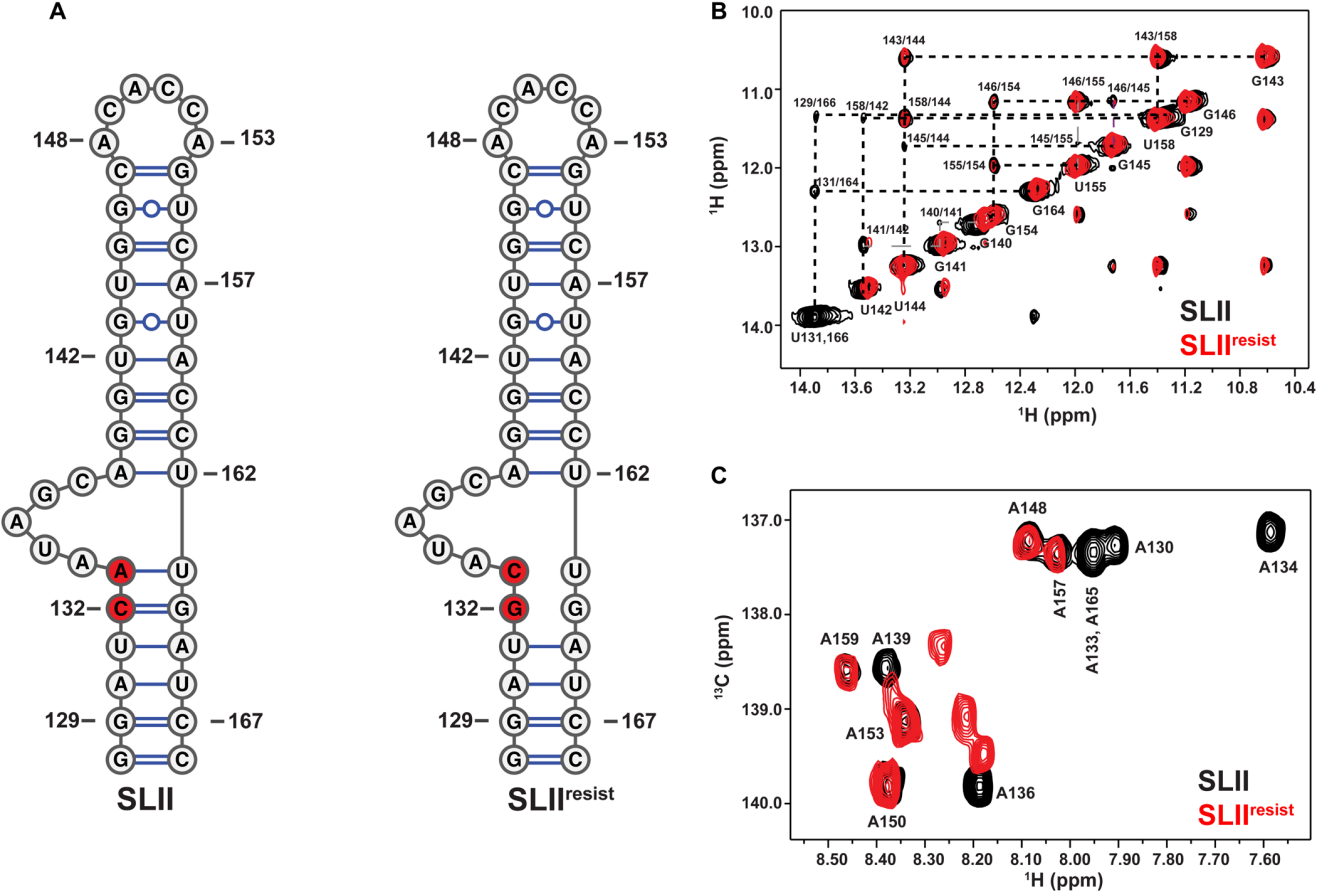
SLII^resist^ folds into a global structure with similar features to that of wild-type SLII. (**A**) Comparison of the experimentally determined SLII (left) structure to that of SLII^resist^ (right). (**B**) Overlay of the ^1^H-^1^H NOESY spectra of SLII (black) and SLII^resist^ (red) reveals that the two RNAs have identical (G/U)NH-(G/U)NH cross peak patterns for nucleotides corresponding to the apical loop and upper helix. NOE cross peaks corresponding to U131 and U166 located in the lower helix of SLII are missing in SLII^resist^ providing evidence that the C132G and A133C mutations change the local structure near the bulge loop. The spectra were recorded at 900 MHz in 10 mM K_2_HPO_4_ (pH 6.5), 20 mM KCl, 0.5 mM EDTA, and 4 mM BME D_2_O buffer at 298 K. (**C**) Overlay of the TROSY HSQC spectra of A(^13^C)–selectively labeled SLII (black) and SLII^resist^ (red) demonstrate that the base stacking arrangements of the two RNAs are similar within the upper apical loop region but differ within the bulge loop. The spectra are centered on the C8-H8 region and were collected in 10 mM K_2_HPO_4_ (pH 6.5 before exchanging in D_2_O), 20 mM KCl, 0.5 mM EDTA, and 4 mM BME D_2_O buffer at 298 K.

We next characterized the (DMA-135)-resistant EV-A71 mutant obtained at passage 9. Following purification of virus from one selected plaque and reverse transcription polymerase chain reaction (RT-PCR) of the 5′UTR, the resulting DNA was sequenced to identify mutations that may confer its resistance to DMA-135. Two mutations that mapped to the bulge loop region of the SLII domain were identified (Fig. 1B). In particular, C132 and A133 were substituted to G132 and C133 in the mutant virus (C132G and A133C). These nucleotides are immediately adjacent to the bulge structure of SLII (hereafter referred to as SLII^resist^; see Figs. 1B and 2A). To evaluate whether the C132G and A133C mutations alone were sufficient to confer drug resistance, we performed site-directed mutagenesis of the wild-type infectious clone cDNA of EV-A71 to introduce the two nucleotide substitutions. Transfection of Vero cells with the resulting EV-A71 mutant RNA, prepared by in vitro transcription, was used to generate mutant virus harboring only the two SLII mutations. Subsequently, SF268 cells were infected with wild-type or mutant virus in parallel, with or without 50 μM DMA-135. Notably, this experiment shows that the C132G- and A133C-engineered mutant virus is refractory to DMA-135 at this concentration compared to wild-type EV-A71 (Fig. 1C). These results unambiguously prove that the bulge loop environment of SLII is the biologically relevant target of DMA-135 and that the mutations identified are sufficient to confer DMA-135 resistance to EV-A71.

### Effects of DMA-135 on IRES-dependent translation in EV-A71 harboring the SLII^resist^ mutations

Building on the identification of the C132G- and A133C-resistant mutations in the SLII IRES domain, we next evaluated whether DMA-135 affected IRES-dependent translation using a dual-luciferase reporter assay with lysates of cells transfected with EV-A71 IRES–driven reporter RNAs. Reporter plasmids harboring the wild-type or resistant 5′UTR (5′UTR^resist^) linked to firefly luciferase served as templates for in vitro synthesis of capped and polyadenylated reporter RNAs, RLuc-(EV-A71/5′UTR)-FLuc and RLuc-(EV-A71/5′UTR^resist^)-FLuc, respectively (see Materials and Methods). The 5′ ORF in both RNAs is Renilla luciferase (RLuc), the translation of which is cap-dependent and thus serves as an internal control. The respective RNAs were transfected into SF268 cells cultured in the absence or presence of DMA-135 (50 μM). The activities of Renilla (RLuc) and Firefly (FLuc) were measured 2 days after transfection. As previously reported, DMA-135 attenuates IRES-dependent translation (FLuc) with no statistically significant effects on cap-dependent translation (RLuc) with the wild-type 5′UTR reporter (Fig. 1D). In particular, FLuc activity declined by 94% with 50 μM DMA-135, while RLuc activity remained constant with or without DMA-135. Conversely, FLuc activity was unaffected by 50 μM DMA-135 with the 5′UTR^resist^-containing reporter RNA. As expected, control RLuc was unaffected by DMA-135, indicating that DMA-135 has no effect on cap-dependent translation. These collective results strongly support that the mechanism by which DMA-135 attenuates IRES-dependent translation is via binding to the bulge loop surface of SLII, as we showed previously by NMR spectroscopy (*15*).

### Changes to the SLII structure induced by the DMA-135 resistant mutations

To better understand how the C132G and A133C mutations in SLII contribute to DMA-135 resistance, we proceeded to study the structural properties of this RNA construct by NMR spectroscopy. We confirmed the secondary structure of SLII^resist^ (Fig. 2A) by comparing its ^1^H-^1^H nuclear Overhauser effect spectroscopy (NOESY) spectrum collected in H_2_O to that of the wild-type construct. Identical NOE cross peaks are observed between imino hydrogens for the upper helices of both wild-type SLII and SLII^resist^ indicating that the C132G and A133C mutations do not perturb the base pairing configuration of the apical loop environment (Fig. 2B). By contrast, the sequential NOEs involving the U131/U166 spin systems observed in the spectrum of wild-type SLII are gone in SLII^resist^, verifying that the resistance mutations change the secondary structure of the bulge proximal region.

Further evidence of localized structural differences in SLII^resist^ relative to wild-type was verified by comparing ^1^H-^13^C heteronuclear single-quantum correlation (HSQC) spectra collected on samples prepared with A(^13^C)–selective labeling. Figure 2C shows an overlay of the C8-H8 region of the ^1^H-^13^C transverse relaxation–optimized spectroscopy (TROSY) HSQC of SLII^resist^ and SLII. The C8-H8 correlation signals belonging to the adenosines of the upper helix (A157 and A159) and the apical loop (A148, A150, and A153) in the SLII^resist^ construct overlay perfectly with those observed in wild-type SLII. The correspondence between the ^1^H and ^13^C chemical shifts of these residues indicates that the C132G and A133C mutations do not substantially perturb base stacking interactions within and nearby the apical loop. By contrast, the NMR signals of A130, A134, A136, A139, and A165—bases within or proximal to the bulge—shift to different positions within the spectrum (note that A133 becomes C133 in SLII^resist^). The different chemical shifts of these residues relative to the wild-type SLII construct is evidence that the mutations change the local physicochemical environment of the bulge. To more precisely evaluate the influence of the C132G and A133C mutations on local base stacking, we compared ^1^H-^1^H NOESY spectra collected in D_2_O on SLII and SLII^resist^ constructs prepared with CU(^2^H),AG(^2^H_3’-5″_) selective labeling. The overlaid spectra show that the C132G and A133C mutations abrogate base stacking within the bulge loop because the A136 to G137 NOE cross peaks observed for SLII are not observable in SLII^resist^ (fig. S1A). The spin system associated with A133 is missing entirely in the SLII^resist^ spectrum as a result of the A133C mutation. The remainder of the NOE cross peaks and patterns are similar between SLII and SLII^resist^. Collectively, the NMR results demonstrate that viral evolution, driven by the selective pressure of DMA-135, leads to a variant with structurally perturbing mutations localized to the bulge loop environment of SLII^resist^.

### Increased structural plasticity of SLII caused by the DMA-135 resistant mutations

**Fig. 3.**
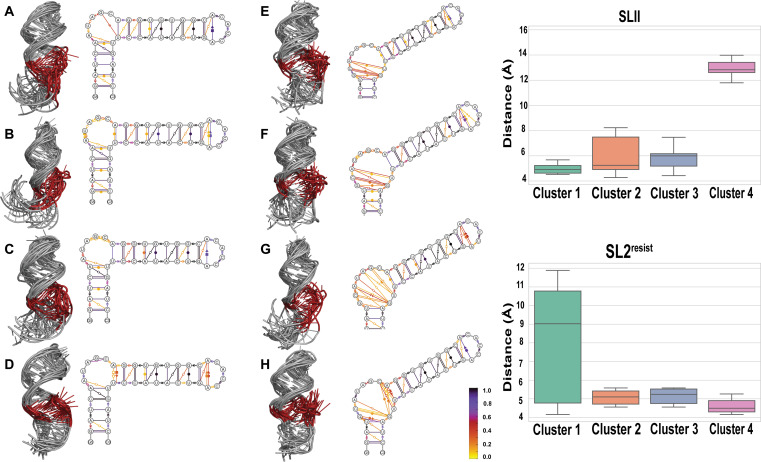
The bulge loop of SLII^resist^ is more conformationally heterogeneous than SLII but both display similar correlated motions at the hnRNP A1 binding site. Representative conformational ensembles of SLII (**A** to **D**) and SLII^resist^ (**E** to **H**). Each cluster contains 10 superimposed structures to highlight the intrinsic conformationally heterogeneity of the RNA molecules. Dynamic secondary structure representations of 100 conformers of SLII and SLII^resist^ are also shown following the Leontis-Westhof classification system. The C132G and A133C mutations lead to an increase in the conformational dynamics of the bulge loop of SLII^resist^ relative to wild type SLII. The box-and-whisker plots show the distribution of distances between the aromatic rings of A136 and G137 in four clusters of SLII and SLII^resist^. The distance distributions reveal that the conformational motions of A136 and G137 are predominantly correlated in both RNA molecules.

RNA structures are known to adopt conformational ensembles that sample different functional and nonfunctional states (*23*, *24*). The SLII bulge loop is the binding site of hnRNP A1 and AUF1, which compete to differentially regulate IRES-dependent translation (*19*). To understand how the C132G and A133C mutations perturbed the SLII conformational landscape with a focus on the bulge loop, we performed 8.6 μs of molecular dynamics (MD) simulations and used Markov state modeling (MSM) to compare the relative conformational ensembles of wild-type SLII and SLII^resist^ (Fig. 3). Time-independent components analysis (TICA) results indicate that the conformational landscapes of SLII and SLII^resist^ are energetically different with the ensemble of SLII^resist^ conformers adopting substantially higher free energy states (fig. S2), indicating that the C132G and A133C mutations increases the overall plasticity of the SLII regulatory axis (Fig. 3). This observation is consistent with the ^1^H-^1^H NOESY spectrum that shows that the U131/U166 imino spins systems are missing in the spectrum collected on SLII^resist^ (Fig. 2B). We next applied MSM to gain insights on the physicochemical features perturbed by comparing the four most populated clusters of SLII and SLII^resist^ extracted from the Perron-cluster cluster analysis distribution of 8.6-μs simulation. MSM clustering revealed that there is a notable difference in bulge topology, indicating that there are distinct conformational changes resulting from the DMA-135–induced mutations, which remove canonical Watson-Crick base pairs and associated hydrogen bonds. Notably, the intermolecular interaction network within the bulge environment (A134 to C138 and U162, U163) consists of more conformational heterogeneous contacts in SLII^resist^ compared to wild-type SLII, suggesting that the C132G and A133C mutations lead to non-nearest neighbor effects.

HnRNP A1 regulates EV-A71 replication in part by binding to A136 and G137 to stimulate IRES-dependent translation. As described above, the activity of IRES elements harboring the C132G and A133C mutations is similar to wild-type levels, indicating that the hnRNP A1 binding site remains functional in SLII^resist^. To understand whether the C132G and A133C mutations change the conformational landscape of the hnRNP A1 binding site, we measured the distribution of the intermolecular distances between the center of the aromatic rings of A136 and G137 for each conformer from the MSM clusters of SLII and SLII^resist^. We used the A136 and G137 intermolecular distance distributions as a proxy of their correlated dynamics given that hnRNP A1 recognizes both to form a functional complex with SLII. Figure 3 shows that the average intermolecular distances between A136 and G137 for three of the four clusters of both SLII and SLII^resist^ is less than 5 Å, whereas both can adopt conformations for which the average intermolecular distance exceeds 9 Å. These observations suggest that the C132G and A133C mutations of SLII^resist^ do not perturb the correlated motions of the hnRNP A1 binding site. In sum, the extended MD simulations reveal that the C132G and A133C mutations increases the conformational heterogeneity of SLII^resist^ relative to SLII without affecting the correlated spatial disposition of the core A136-G137 hnRNP A1 binding site.

### Binding of SLII^resist^ by DMA-135 and inability to induce formation of a ternary complex

**Fig. 4.**
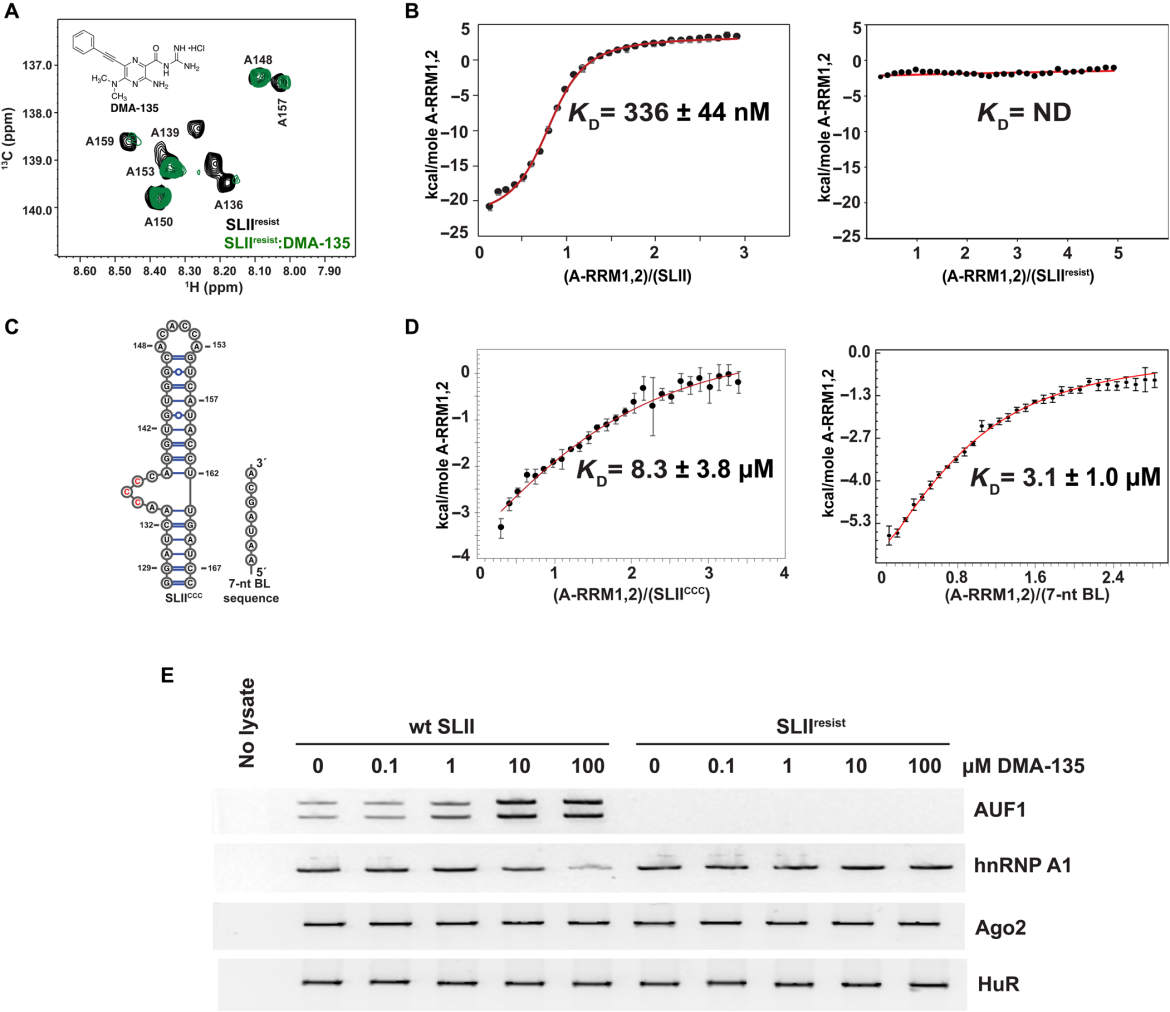
The sequence and structure of the SLII bulge loop facilitates intermolecular interactions. (**A**) Single-point TROSY HSQC titration of A(^13^C)–selectively labeled SLII^resist^ with DMA-135 at a fivefold excess. The black correlation peaks correspond to free SLII^resist^ and the red to the (DMA-135)-SLII^resist^ complex. The spectra were collected at 900 MHz in 10 mM K_2_HPO_4_ (pH 6.5 before exchanging in D_2_O), 20 mM KCl, 0.5 mM EDTA, and 4 mM BME D_2_O buffer at 298 K. (**B**) Calorimetric titrations of A-RRM1,2 into SLII (left) and SLII^resist^ (right). The A-RRM1,2-SLII data were fit to a 1:1 stoichiometric binding model in Affinimeter (*39*). ND, not determined. Reported values for *K*_D_ and corresponding SD are from triplicate experiments. (**C**) RNA constructs used to assess determinants of specific and high-affinity AUF1-SLII interactions. Left, SLII^CCC^ replaces the phylogenetically conserved UAG bulge motif with CCC. Right, a 7-nt oligonucleotide that mimics the SLII bulge loop sequence with adjacent nucleotides. (**D**) Calorimetric titrations of A-RRM1,2 into SLII^CCC^ (left) and the 7-nt oligonucleotide (right). The A-RRM1,2-SLII^CCC^ data were fit to a 1:1 stoichiometric binding model in Affinimeter (*39*). Reported values for *K*_D_ and corresponding SD are from triplicate experiments. (**E**) Protein-biotinylated RNA pull-down experiments were performed to evaluate the influence of DMA-135 on the interaction between AUF1 and SLII^resist^. Biotinylated SLII and SLII^resist^ RNAs were transfected into SF268 cells. Cells were cultured with increasing concentrations of DMA-135. Cell lysates were used for pull-down assays of SLII-associated proteins AUF1, hnRNP A1, Ago2, and HuR and detection by Western blotting.

Since the C132G and A133C mutations change the local bulge loop structure, we evaluated whether DMA-135 retains binding affinity for SLII^resist^. Using the A(^13^C) selectively labeled SLII^resist^ construct, we performed a single-point ^1^H-^13^C TROSY HSQC titration, as described previously (*15*, *25*). Given the labeling strategy, we can assess the extent to which DMA-135 interacts with specific surfaces on SLII^resist^ because the degree of NMR signal perturbations is a proxy for complex formation (*15*, *25*). Figure 4A shows the effects of the addition of excess (5:1) DMA-135 on the C8-H8 correlation signals of SLII^resist^. The spectrum shows that several of the correlation peak exchanges broaden in the presence of excess DMA-135; however, the signals that overlap with nucleotides from the apical loop and upper helix of wild-type SLII remain mostly unperturbed (A148, A150, A153, and A157). This observation indicates that although the local structure of the bulge motif has changed, DMA-135 can still recognize it as a binding surface. We verified that DMA-135 binds site-specifically to SLII^resist^ by collecting a ^1^H-^1^H NOESY spectrum of the complex in D_2_O (fig. S1B). SLII^resist^ was prepared with CU(^2^H),AG(^2^H_3’-5″_) selective labeling to which unlabeled DMA-135 was added at a 5:1 molar ratio. The overlay of the ^1^H-^1^H NOESY spectra of the free and DMA-135 bound forms of SLII^resist^ shows that several NOE cross peaks observed for free SLII^resist^ disappear or shift to different positions in the complex; however, NOE patterns that correspond to residues located in the upper helix are mostly preserved (fig. S1B). Notably, inter-NOEs between the methyl groups on DMA-135 and residues from SLII^resist^ are also observed in the spectrum (fig. S1B). Together, these data provide NMR evidence that DMA-135 binds to a specific surface on SLII^resist^ via a mechanism that does not substantially perturb the upper helical geometry.

Because DMA-135 can still interact with the bulge loop environment, we next decided to test whether it can allosterically increase the affinity of AUF1 for SLII^resist^ like it does for the wild-type SLII (*15*). We posited that the mechanism of action (functional specificity) of DMA-135 is its ability to shift the AUF1-SLII equilibrium to favor suppression of IRES-dependent translation. Figure 4B shows a calorimetric titration of the RNA binding domain of AUF1 (A-RRM1,2) titrated into wild-type SLII and SLII^resist^. As previously observed, the thermogram shows that A-RRM1,2 binds SLII as a specific 1:1 complex with high affinity [dissociation constant (*K*_D_) = 336 ± 44 nM]. By contrast, the thermogram of A-RRM1,2 titrated into SLII^resist^ is flat with no change in the total binding enthalpy over the course of the titration. This observation indicates that C132 and/or A133 are determinants of high-affinity A-RRM1,2-SLII recognition.

To investigate the sequence and structure contributions further, we performed calorimetric titrations of A-RRM1,2 with a laboratory-derived SLII mutation where the central UAG motif is mutated to CCC (SLII^CCC^) and a synthetic 7-nt oligo (5′-AAUAGCA-3′) that mimics the native bulge sequence and adjacent A133 and A139 nucleotides (Fig. 4C). A-RRM1,2 binds SLII^CCC^ very weakly (*K*_D_ = 8 ± 3 μM) and nonspecifically as determined by the shape of the binding isotherm relative to the wild-type SLII control titration (Fig. 4D). This observation is consistent with prior results where we were unable to detect AUF1 in pull-down assays using a biotinylated SLII^CCC^ RNA (*15*). By comparison, the binding affinity of A-RRM1,2 for the 7-nt oligo is also weak (*K*_D_ = 3 ± 1 μM) but slightly tighter than that determined for SL^CCC^. When interpreted collectively, the titrations of A-RRM1,2 with wild-type SLII, SLII^resist^, SLII^ccc^, and the 7-nt oligo reveal the importance of the bulge loop structure, its sequence, and the contributions of the A133-A134 dinucleotide to high-affinity binding.

We decided to test whether the physicochemical property of SLII^resist^ to no longer bind AUF1 productively also occurred in a more biological context using an assay where SLII^resist^ was biotinylated and used to pull down endogenous AUF1. Biotinylated SLII^resist^ and wild-type SLII RNAs were transfected into SF268 cells in the presence of various concentrations of DMA-135. Twelve hours after transfection, cells were lysed, and the complexes of cellular proteins bound to SLII^resist^ and SLII wild-type were captured using streptavidin-sepharose conjugated beads; levels of captured AUF1 were assessed by Western blot; hnRNP A1 served as a control. Consistent with the calorimetric results, we were unable to detect a complex between SLII^resist^ and endogenous AUF1, whereas the AUF1-SLII wild-type complex readily increased with DMA-135 concentration, as we showed previously (Fig. 4E) (*15*). By contrast, binding by hnRNP A1 to SLII^resist^ was readily detected with both the wild-type and resistant SLII (also see next section below), although there was a slight decrease in binding by hnRNP A1 to native SLII at the highest DMA-135 concentration tested, 100 μM (Fig. 4E). As a control, we examined two additional ITAFs that are essential for IRES activity, RISC subunit Ago2 and AU-rich element mRNA-stabilizing protein HuR (*26*). Like hnRNP A1, both proteins associated with SLII regardless of DMA-135 concentration. Moreover, they bound SLII^resist^ as well, and binding was unaffected by DMA-135 (Fig. 4E). Collectively, these results solidify the allosteric mechanism of DMA-135, and it shows that EV-A71 can evolve to shift the binding affinity of AUF1 for SLII when under selective pressure.

### Selective effects of DMA-135 on AUF1-RNA interactions

**Fig. 5.**
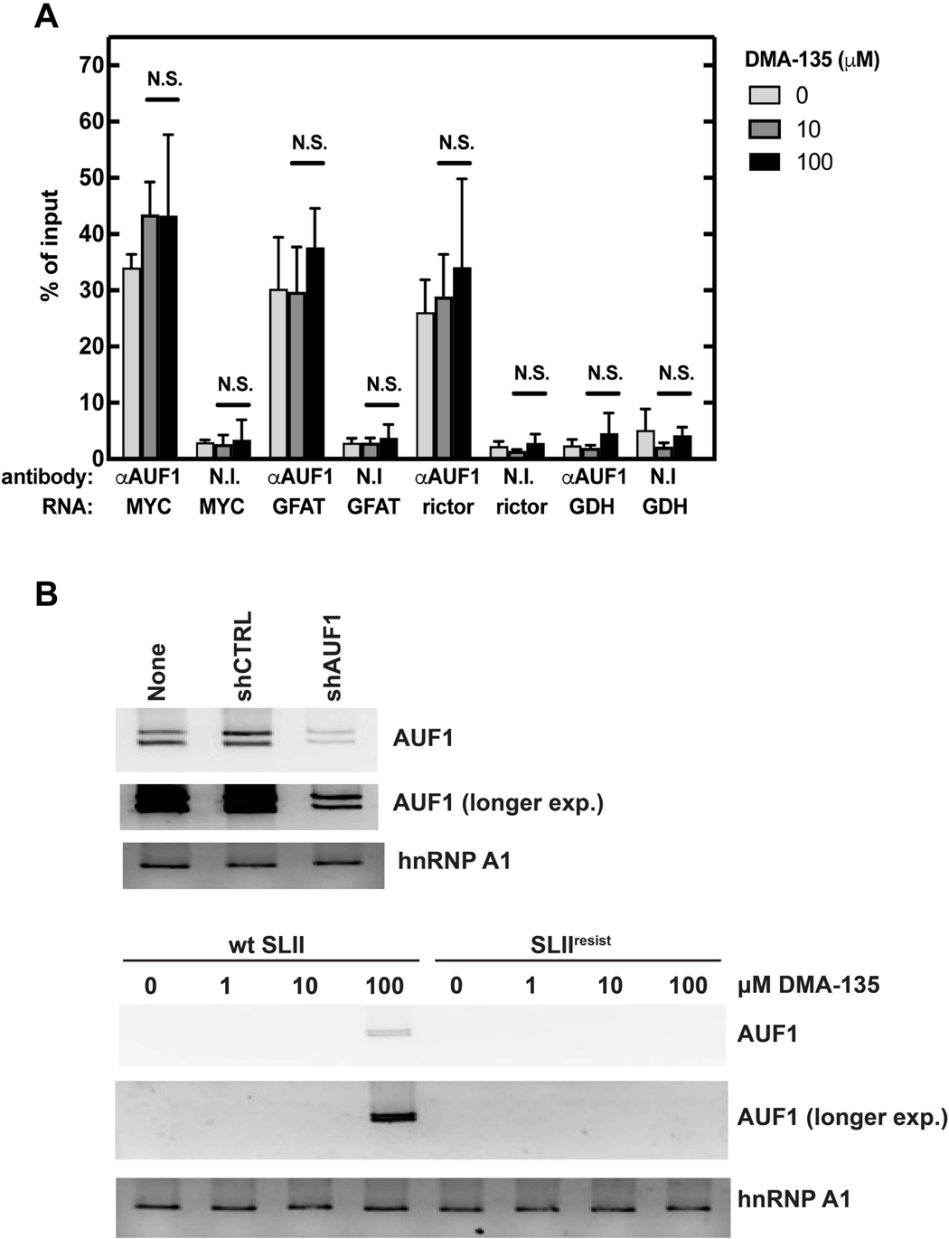
Analyses of the effects of DMA-135 on AUF1-RNA interactions. (**A**) DMA-135 does not globally affect AUF1-mRNA association. RIP assays of endogenous protein-RNA complexes were used to assess effects of DMA-135 on AUF1 association with select target mRNAs. For the original experiment (*15*), SF268 cells were transfected with luciferase reporter RNAs harboring either the wild-type EV-A71 5′UTR or with a CCC-bulge mutant SLII. Cells were cultured with various concentrations of DMA-135. Lysates were immunoprecipitated with nonimmune (N.I.) or AUF1 antibody. The same input and immunoprecipitated materials from the earlier work (*15*) were reanalyzed by qRT-PCR for AUF1 association with target mRNAs encoding MYC, GFAT1, and rictor proteins versus DMA-135 concentration. Mean values ± SDs from three independent experiments are shown. *P* values were determined by unpaired two-tailed Student’s *t* test with *P* < 0.05 considered statistically significant. N.S., not significant. (**B**) Effects of DMA-135 on AUF1-SLII RNA interactions following AUF1 knockdown. (Top) Knockdown of AUF1. SF268 cells were either not transfected (none) or transfected with plasmids expressing a control shCTRL or an shRNA targeting all four AUF1 isoform mRNAs (shAUF1). After two days, AUF1 knockdown was assessed by Western blot; hnRNP A1 was an internal control. Two exposures of the AUF1 immunoblot are shown. (Bottom) Effects of AUF1 knockdown and DMA-135 on SLII RNA-protein interactions. Cells transfected with shRNA-expressing plasmids for two days were then transfected with biotinylated SLII or SLII^resist^ RNAs. After four hours, cells were cultured with the indicated concentrations of DMA-135 for 12 hours. Cell lysates were incubated with streptavidin-sepharose and purified, biotinylated RNA-protein complexes were analyzed by Western blot for AUF1 and hnRNP A1. Two exposures of the AUF1 immunoblot are shown. N.S., not significant.

As described previously, DMA-135 acts to allosterically enhance binding of AUF1 to SLII via stabilization of a ternary complex of (DMA-135)-SLII-AUF1 (*15*). The net effect was a DMA-135 concentration–dependent increase in the overall amount of SLII molecules bound by AUF1. This was reflected as a DMA-135–dependent increase in SLII-AUF1 complexes in cells as assayed by RNP-immunoprecipitation (RIP) assays using AUF1 antibody, followed by quantitative RT-PCR (qRT-PCR) to quantify captured RNA (see Materials and Methods). However, it is not known whether the DMA-135 effect is specific for SLII or whether it extends to endogenous, cellular AUF1 target mRNAs. Hence, qRT-PCR analyses of the originally purified RNA samples from RIPs performed with nonimmune or AUF1 antibody were extended to include three randomly chosen cellular mRNAs bound by AUF1: proto-oncogene *MYC*, *GFAT1* (glutamine fructose-6-phosphate amidotransferase 1), and the mammalian target of rapamycin complex 2 (mTORC2) component, *rictor* (*27*). Glyceraldehyde-3-phosphate dehydrogenase (*GAPDH*) mRNA, which is not an AUF1 target, served as a negative control. The qRT-PCR analyses indicated that DMA-135 had no statistically significant effect on the amount of *MYC*, *GFAT1*, or *rictor* mRNA bound by AUF1 with the DMA-135 concentrations tested: 0, 10, and 100 μM (Fig. 5A). (*GAPDH* mRNA was present at background levels, i.e., similar levels to RIPs with nonimmune antibody). In other words, DMA-135 did not increase levels of AUF1-mRNA target complexes. By contrast, DMA-135 had statistically significant effects on the levels of AUF1-SLII complexes at 1, 10, and 100 μM DMA-135 in the original experiments [*P* < 0.01 to 0.001, *N* = 3; see figure 7C in (*15*)]. We conclude that the DMA-135 effects on AUF1-RNA associations are not global but, rather, present specificity toward EV-A71 SLII RNA.

### Effects of reduced AUF1 expression on antiviral activity of DMA-135

Given the importance for the effect of DMA-135 on virus replication, we tested the outcome of reducing AUF1 abundance by RNA interference–induced knockdown. SF268 cells were either not transfected or transfected with plasmids expressing a control, scrambled short hairpin RNA (shRNA, shCTRL) or shRNA directed against all four *AUF1* isoform mRNAs (shAUF1). Cells were then infected with EV-A71, and cultures were incubated with 0, 10, or 100 μM DMA-135. Virus titers were determined 24 hours later. Western blot analysis showed that AUF1 knockdown was ~80% (Fig. 5B, top; compare the short and long exposures of the AUF1 immunoblot). As expected, reduced AUF1 levels led to a 10-fold increase in virus titer compared to the control knockdown [1.2 × 10^9^ pfu/ml versus 1.2 × 10^8^ pfu/ml, respectively; (*19*)]. However, 10 μM DMA-135 led to three and four log_10_ reductions in titers with control and AUF1 knockdown cells, respectively. Virus titers for both were <10 pfu/ml with control and AUF1 knockdown cells at 100 μM DMA-135.

This raised the possibility that DMA-135 may nonetheless increase the binding of residual AUF1 to SLII, maintaining drug activity. To test this, cells transfected with shAUF1-expressing plasmid were transfected with biotinylated forms of SLII or SLII^resist^ RNAs and cultured with 0 to 100 μM DMA-135 for 12 hours. Pull-down assays were performed with cell lysates using streptavidin-sepharose, and AUF1 and hnRNP A1 were detected by Western blot (Fig. 5B, bottom). While lower levels of AUF1 captured upon AUF1 knockdown was expected, it is readily detected at 100 μM DMA-135, especially upon longer exposure of the AUF1 immunoblot (Fig. 5B, bottom; compare the short and long exposures of the AUF1 immunoblot). AUF1 levels at 1 and 10 μM, DMA-135 appear to be below the limit of detection. No detectable AUF1 binding was observed with SLII^resist^ RNA at any DMA-135 concentration, while binding by hnRNP A1 was relatively constant across all conditions tested. These results are consistent with those in the biotinylated RNA-protein pull-down assays (Fig. 4E). We conclude that residual AUF1 may be sufficiently abundant enough to maintain the antiviral activity of DMA-135.

### Effects of DMA-135 on the SLII-hnRNP A1-AUF1 regulatory axis

**Fig. 6.**
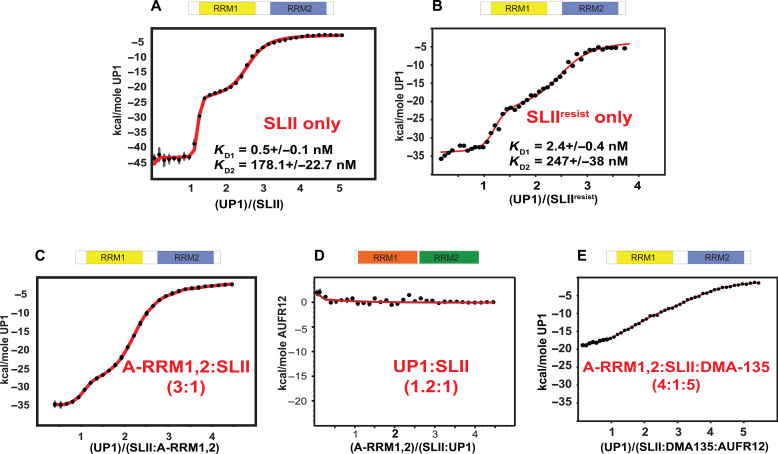
AUF1 and hnRNP A1 compete for the bulge loop surface on SLII. (**A** to **E**) Comparative calorimetric titrations of the RNA binding domains of hnRNP A1 (UP1, yellow/blue rendering) and AUF1 (A-RRM1,2, orange/green rendering) demonstrate that the two proteins directly compete for the bulge loop surface on SLII and that the degree of the competition is modulated by DMA-135. Thermodynamic parameters derived from fits to a two-independent sets of site model using Affinimeter (*39*) are reported for the UP1-SLII and UP1-SLII^resist^ titration only. Thermodynamic parameters are not reported for the other titrations given the complexity of the competitive binding equilibria. Qualitative comparisons demonstrate that the thermodynamic parameters are observably different for UP1 titrated into preformed complexes of AUF1-SLII and AUFI-SLII-(DMA-135) as well as AUF1 titrated into a preformed complex of UP1-SLII.

SL II functions as a regulatory axis during EV-A71 replication by modulating the efficiency of IRES-dependent translation. Several ITAFs and a vsRNA1 bind to SLII to differentially control IRES activity, and DMA-135 overrides these regulatory mechanisms to repress viral translation (*11*, *15*–*21*). To better understand how the physicochemical properties of SLII contribute to EV-A71 biology, we carried out a series of calorimetric titrations using the RNA binding domains of hnRNP A1 (UP1) and AUF1 (A-RRM1,2). Titrations of UP1 into SLII^resist^ resulted in a biphasic isotherm similar to that previously observed for wild-type SLII (Fig. 6). Fitting of the processed data to a two-independent site binding model reveals that both events are characterized by nanomolar affinities (*K*_D1_ = 2.4 ± 0.4 nM, *K*_D2_ = 247 ± 38 nM), which are on the same order of magnitude as those measured here for wild-type SLII (*K*_D1_ = 0.5 ± 0.1 nM, *K*_D2_ = 178.1 ± 22.7 nM). This result is consistent with the data described above where hnRNP A1 retains binding affinity for SLII^resist^ (±DMA-135) within a biological context (Fig. 4E). These collective observations indicate that the C132G and A133C mutations do not substantially impair the hnRNP A1-SLII arm of the regulatory axis, allowing hnRNP A1 to continue to stimulate translation during the earliest stages of viral replication.

Given that hnRNP A1, but not AUF1, can still bind SLII^resist^, we reasoned that DMA-135 modulates the SLII regulatory axis by affecting the extent to which these two proteins compete for the bulge loop. To that end, we performed competitive calorimetric titrations of A-RRM1,2 and UP1 into SLII alone or pre-bound in various complexes (Fig. 6 and fig. S3). As described previously (*15*), A-RRM1,2 binds SLII as a specific 1:1 complex with a *K*_D_ ~330 nM. We further probed the specificity of the SLII-(A-RRM1,2) interaction here by resolving the complex by size exclusion chromatography (SEC) and mapping the binding interface by NMR (fig. S4). The data show that A-RRM1,2 binds to the bulge loop of SLII and that the complex is stable throughout the course of the SEC run. Despite the stability of the SLII-(A-RRM1,2) complex, binding is undetectable when A-RRM1,2 is titrated into a preformed UP1:SLII (1.2:1) complex (Fig. 6D). By comparison, UP1 can still bind SLII when in the presence of excess A-RRM1,2, albeit with a characteristically different biphasic isotherm compared to the control titration (Fig. 6, A and C). The initial transition that corresponds to UP1 binding the bulge loop has a shallower inflection and a lower total change in binding enthalpy (Fig. 6, A and C). Conversely, the portion of the isotherm that reflects binding of UP1 to the apical loop is primarily unchanged. These results show that hnRNP A1 and AUF1 directly compete for the bulge loop surface and that hnRNP A1 can displace AUF1, which is consistent with the more than 500-fold difference in their binding affinities for the bulge loop.

Next, we decided to see whether DMA-135 changes the binding properties by titrating UP1 into a preformed (DMA-135)-SLII-(A-RRM1,2) ternary complex. UP1 no longer efficiently displaces A-RRM1,2 from the bulge as determined by the shape of the isotherm and change in total binding enthalpy relative to the binary (A-RRM1,2-SLII) titration (Fig. 6E). These collective results agree with the antiviral mechanism of action of DMA-135, which is to decrease cap-independent translation by stabilizing the repressive AUF1-SLII complex. DMA-135 shifts the (hnRNP A1/AUF1)–SLII regulatory axis just enough such that viral protein synthesis becomes inhibited during the EV-A71 replication cycle.

### Reduced DMA-135–dependent inhibition of EV-D68 replication

**Fig. 7.**
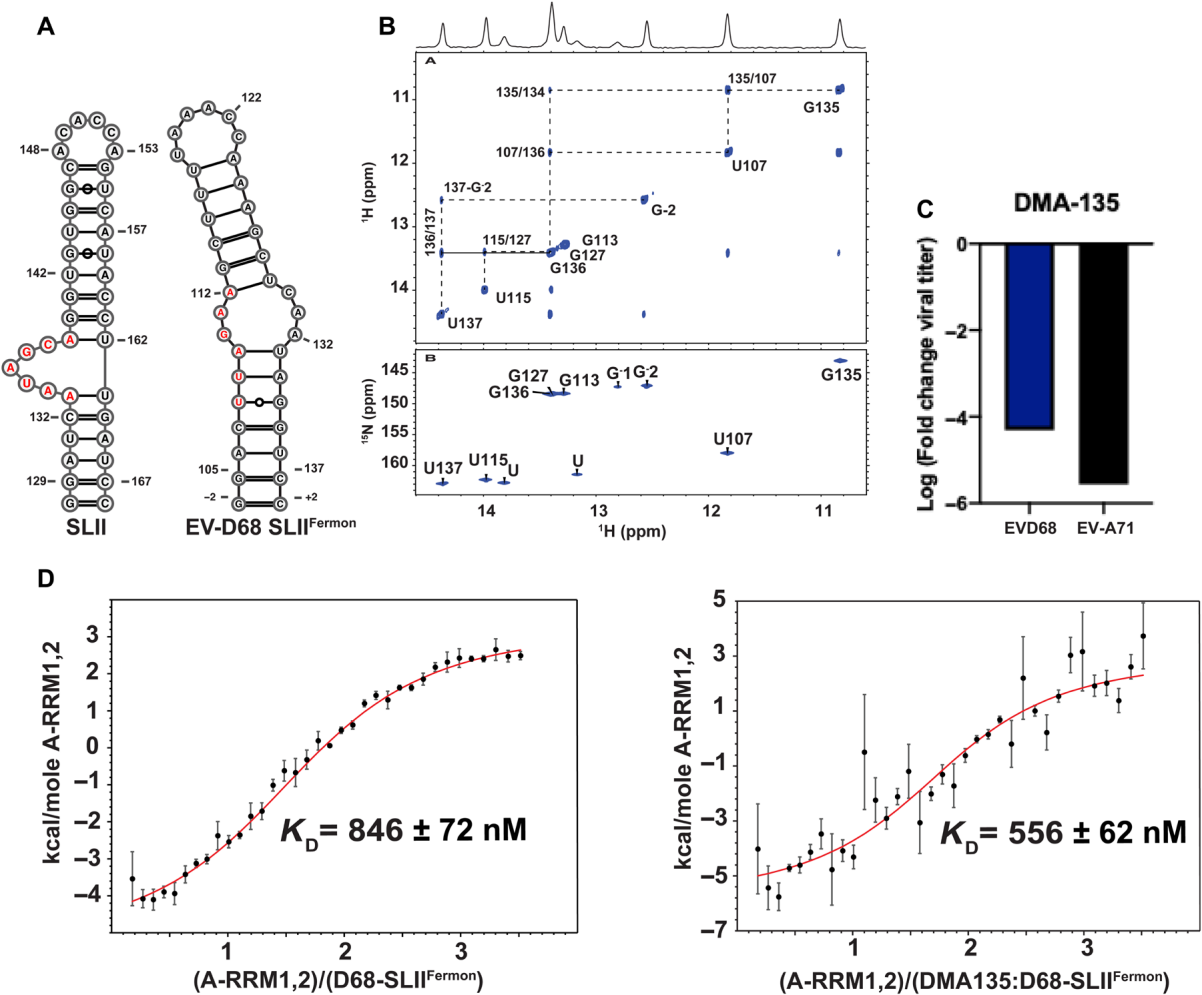
Assessment of the antiviral capacity of DMA-135 against EV-D68. (**A**) Comparison of the experimental secondary structure of SLII from EV-A71 (left) to that predicted for EV-D68 (right). (**B**) Top, imino region of the ^1^H-^1^H NOESY (700 MHz and *t*m = 200 ms) spectrum of EV-D68 SLII collected in 10 mM K2HPO4 (pH 6.5), 20 mM KCl, 0.5 mM EDTA, and 4 mM TCEP with 10% D2O, at 283 K. The vertical and horizontal dashed lines trace the NOE stacking pattern for each stable helical region demonstrating that EV-D68 SLII^Fermon^ is an independently folded domain. Bottom, ^1^H-^15^N HSQC collected in the buffer condition of 10 mM K2HPO4 (pH 6.5), 20 mM KCl, 0.5 mM EDTA, and 4 mM TCEP with 10% D2O, at 283 K, confirms the^1^H-^1^H NOESY assignments. (**C**) Comparison of the antiviral activity of DMA-135 against EV-A71 (50 μM DMA-135) and EV-D68 (100 μM DMA-135). Log_10_ values of fold change in virus titers compared to the dimethyl sulfoxide (vehicle) control are shown. (**D**) Calorimetric titrations of A-RRM1,2 into (left) EV-D68 SLII^Fermon^ and (right) a preformed (1:5) SLII^Fermon^-(DMA-135) complex. The titration data were fit to a 1:1 stoichiometric binding model in Affinimeter (*39*). Reported values for *K*_D_ and corresponding SD are from triplicate experiments.

To better understand the functional specificity of DMA-135, we decided to test whether it can also inhibit the related EV-D68. We selected the Fermon strain of EV-D68 for this study, which has a predicted SLII structure (SLII^Fermon^) consisting of an internal loop instead of a bulge, and a structurally similar apical loop to that of EV-A71 SLII (Fig. 7A). We confirmed the base pair composition of the SLII^Fermon^ structure by two-dimensional (2D) NMR spectroscopy (Fig. 7B). Sequential and long-range (G/U)NH-(G/U)NH NOE cross peaks can be traced for the internal base pairs of the lower and upper helices verifying that SLII^Fermon^ adopts an overall topology consistent with the predicted structure (Fig. 7B). Addition of 100 μM of DMA-135 to Vero cells infected with EV-D68 [multiplicity of infection (MOI) = 1] reduced viral titers by approximately four orders of magnitude relative to the vehicle (dimethyl sulfoxide) control (Fig. 7C, left). This suggests that the slight differences in the SLII structures (Fig. 7A) may account for the lower efficacy of DMA-135 for EV-D68 relative to EV-A71, which decreased almost six orders of magnitude at 50 μM DMA-135 compared to the vehicle control (Fig. 7C, right). As a further probe of the structural differences, we performed calorimetric titrations of A-RRM1,2 into SLII^Fermon^. The sequence composition of the 5′-half (putative location of the AUF1 binding site) of the internal loop that aligns with the bulge loop (AAUAGCA) of EV-A71 SLII is UUAGAA. A-RRM1,2 binds to SLII from EV-D68 with substantially weaker affinity (*K*_D_ = 846 ± 72 nM) compared to EV-A71 (Fig. 7D). Notably, DMA-135 has a modest effect on the binding affinity (*K*_D_ = 556 ± 62 nM) when A-RRM1,2 is titrated into a preformed (DMA-135)-SLII^Fermon^ complex at a 5:1 molar ratio. We verified that DMA-135 binds with specificity to SLII^Fermon^ by performing a single-point ^1^H-^13^C TROSY HSQC titration as described above. Figure S5 shows that seven of the adenosine C8-H8 correlation peaks are exchanged, broadened upon addition of fivefold excess of DMA-135. SLII^Fermon^ contains 13 adenosines of which six are within or directly adjacent to the internal loop. Thus, we conclude that DMA-135 may also inhibit EV-D68 by binding to the internal loop of SLII. However, additional functional and viral evolution studies are needed to better understand whether the mechanism of action of DMA-135 on EV-D68 replication is similar to that observed here for EV-A71.

## DISCUSSION

Viral evolution under selective pressures of small molecules with capacity to inhibit replication offers opportunities to elucidate mechanisms of action and to reveal previously unknown biology. For small molecules that target viral RNA structures, pressure-driven evolution can also inform on determinants of functional specificity even within a background of potentially nonproductive binding events. Thus, viruses are unique model systems to calibrate principles of small-molecule–RNA interactions and to understand the contributions of RNA structures to biological function. Here, we harnessed the power of viral evolution to reveal the cellular mechanism of the antiviral DMA-135 and to characterize host-virus complexes that differentially regulate EV-A71 translation.

Positive-strand RNA viruses, like EV-A71 and EV-D68, use the same genomic template for translation and viral RNA synthesis (*8*, *11*, *13*, *22*). Following infection, translation proceeds in the cytoplasm via internal loading of the ribosome onto the IRES. The efficiency by which ribosomes are recruited to the IRES determines the frequency by which the viral life cycle transitions from early to late stages because viral protein products are necessary to synthesize nascent genomic RNA and to form infectious virions. Nevertheless, high levels of viral proteins are cytotoxic to the cell, so positive-strand RNA viruses resolve this intrinsic dichotomy by differentially regulating IRES levels via multiple and redundant mechanisms.

Although the complete contributions of the IRES structures of EV-A71 are unknown, the SLII domain is a pivotal regulatory element that coordinates multiple protein-RNA and RNA-RNA interactions to fine-tune viral polyprotein synthesis (*11*, *15*–*21*). Two such interactions include the recruitment of the host RBPs, hnRNP A1, and AUF1. These proteins compete for SLII to either stimulate (hnRNP A1) or repress (AUF1) IRES-dependent translation. Genetically introduced mutations that abrogate the SLII-protein regulatory axis robustly restrict EV-A71 replication by attenuating IRES activity, demonstrating the significance of conserved nucleotide epitopes to viral fitness (*16*, *21*).

The observation that DMA-135 resistance was conferred via noncompensatory mutations of C132G and A133C (part of the AUF1-binding site) suggests that the virus naturally exerts less pressure on repressing translation as opposed to stimulating it. hnRNP A1 (stimulator) retains binding affinity for SLII^resist^ but AUF1 (repressor) is unable to form a detectable complex with the resistant RNA. Hence, EV-A71 escaped sensitivity to DMA-135 by evolving resistant mutations that change the repressive AUF1 arm of the SLII regulatory axis. Furthermore, the activity of the IRES containing SLII^resist^ was comparable to that of the wild-type IRES with or without DMA-135, providing further evidence that inhibition proceeds via selective modulation of the repressive AUF1-SLII complex.

**Fig. 8.**
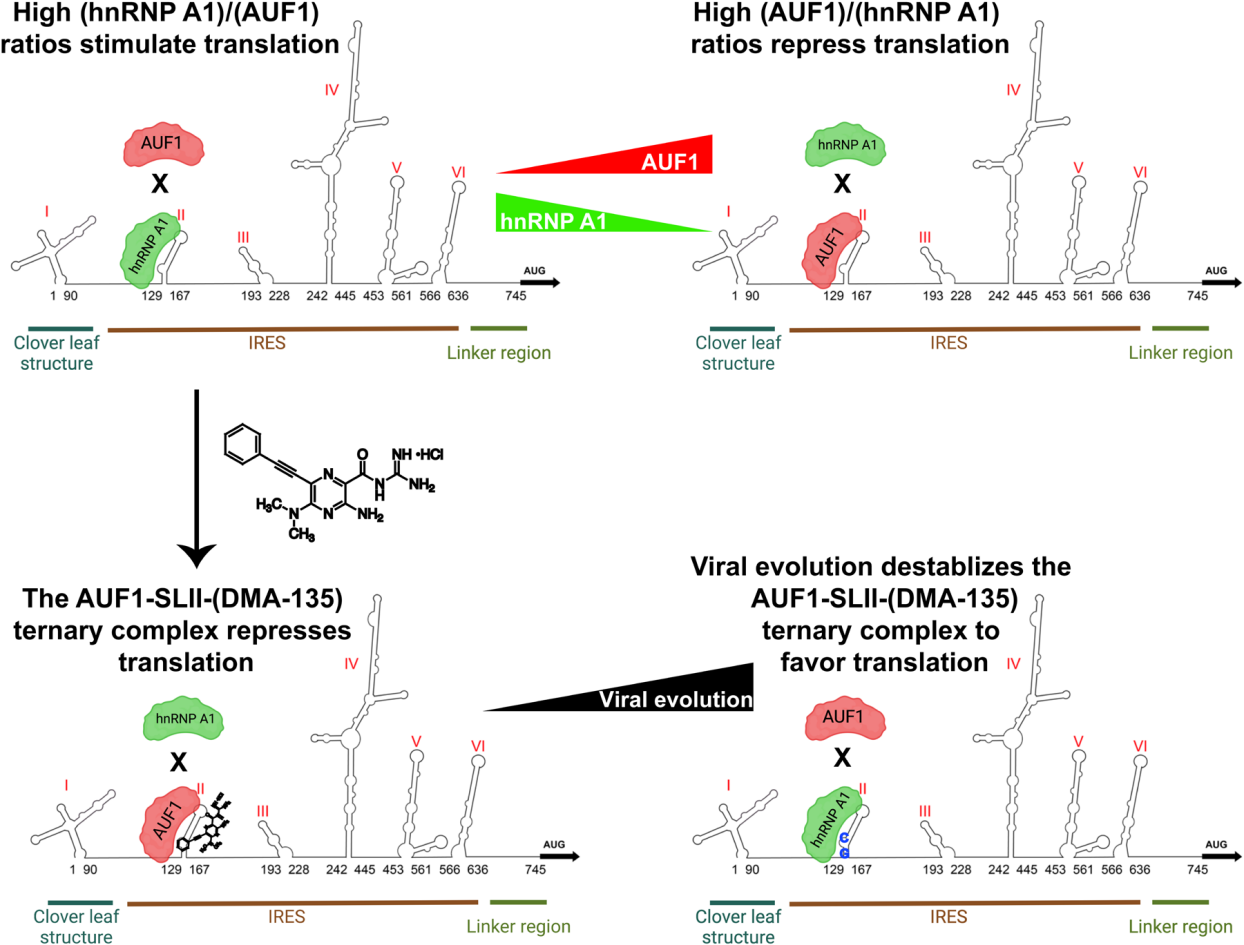
The EV-A71 SLII-hnRNP regulatory axis is under thermodynamic control. An illustration of a conceptual model to interpret the different mechanisms by which the SLII IRES domain coordinates a subset of molecular interactions to in turn modulate translational output and viral replication efficiency. (**Top**) under normal conditions of viral infection, hnRNP A1 and AUF1 compete for the bulge loop of SLII to stimulate or repress viral replication, respectively. Factors that change the relative abundance of hnRNP A1 or AUF1 can shift the SLII regulatory axis toward a repressive or stimulatory direction. (**Bottom**) DMA-135 shifts the SLII-hnRNP regulatory axis by stabilizing a ternary complex with AUF1 and SLII to in turn reduce hnRNP A1’s binding capacity. Viral evolution selects for mutations in the AUF1 binding surface on SLII to shift the regulatory axis back toward favoring translation.

Under non–(DMA-135) conditions, hnRNP A1 alone is able to displace AUF1 pre-bound to SLII; however, it cannot efficiently displace AUF1 when AUF1 is part of a ternary complex with SLII and DMA-135 (Fig. 6E). By comparison, AUF1 cannot displace hnRNP A1 when it is already bound to SLII (Fig. 6D). These observations imply that the SLII regulatory axis is under thermodynamic control, and mechanisms that modulate the relative SLII-protein–binding affinities tune the IRES activity (Fig. 8). In support of this concept, DMA-135 dose-dependently decreases IRES-dependent translation by increasing the affinity of AUF1 for SLII (*15*). Although DMA-135 retains affinity for SLII^resist^, it can no longer functionally inhibit the resistant virus because the A133C mutation changes part of the AUF1 binding epitope to in turn reduce its binding capacity. When interpreted collectively, the functional mechanism of DMA-135, as validated through viral evolution, is to shift the regulatory axis toward the IRES-repressive SLII-AUF1 arm.

We also demonstrated that DMA-135 can inhibit the related EV-D68 virus, albeit ~100-fold less efficiently compared to EV-A71. Comparison of the structural and biophysical properties of the SLII domains from the two viruses offers insights into the relative differences in DMA-135 efficacy. The SLII domain from EV-A71 consists of a conserved 5-nt bulge loop with adjacent AU base pairs. The sequence of the loop environment is 5′-AAUAGCA-3′, which matches the consensus motifs for AUF1 and hnRNP A1 (*28*–*30*). By comparison, SLII from the EV-D68 Fermon strain studied here includes an internal loop with a 5′-UUAGAA-3′ motif that best aligns with the sequence of EV-A71. We showed that AUF1 can bind SLII^Fermon^ as a specific and 1:1 complex, albeit ~3-fold weaker than it forms a complex with SLII from EV-A71. Furthermore, DMA-135 has a modest, yet measurable, influence on the binding affinity of the AUF1-SLII^Fermon^ complex. Together, the data indicate that the potency of DMA-135 against EV-A71 relates to its ability to allosterically recruit AUF1 to an optimal 5′-AAUAGCA-3 bulge loop sequence.

In sum, the work presented here reveals the power of viral evolution to further elucidate functional mechanisms of small molecules with therapeutic capacity against RNA structures. We described the notable observation that that an RNA-targeting small molecule can induce pressure-driven evolution to select for a drug-resistant virus with mutations that map directly to the cellular RNA binding site. The work also demonstrates how small molecules can be deployed as chemical biology reagents to interrogate protein-RNA biological interfaces. We believe that such tools will have a broad range of utilities to better understand protein-RNA networks, including those found in phase separated compartments.

## MATERIALS AND METHODS

### RNA preparation

All SLII constructs used in this study were prepared by in vitro transcription using recombinant T7 polymerase that was overexpressed and purified from BL21 (DE3) cells. Synthetic DNA templates corresponding to the EV-A71 2231 and EV-D68 isolates, or mutant constructs were purchased from Integrated DNA Technologies (Coralville, IA). Transcription reactions were performed using standard procedures and consisted of 3 to 6 ml of reaction volumes containing unlabeled ribonucleotide triphosphates (rNTPs) or (C^13^/N^15^)–labeled rNTPs. Following synthesis, samples were purified to homogeneity by denaturing polyacrylamide gel electrophoresis (PAGE), excised from the gel, electroeluted, and desalted via exhaustive washing of the samples with ribonuclease-free water using a Millipore Amicon Ultra-4 centrifugal device. Samples were annealed by heating at 95°C for 2 min and flash-cooled on ice. Samples were subsequently concentrated and exchanged into 10 mM K_2_HPO_4_ (pH 6.5) and 20 mM KCl, 4 mM tris(2-carboxyethyl)phosphine (TCEP), and 0.5 mM EDTA using a Millipore Amicon Ultra-4 centrifugal filter device. The concentration of the samples was determined using the respective RNA theoretical molar extinction coefficient, and NMR samples ranged from 0.1 to 0.2 mM at 200 μl.

### Protein purification

The AUF1-RRM1,2 (residues 70 to 239 correspond to the p37 isoform) protein construct used in this study was subcloned into a pMCSG7 vector and subsequently overexpressed as an N-terminal (His)_6_–tagged fusion protein in BL21 (DE3). Cells were grown to an optical density at 600 nm of ~1.0 at 37°C and induced with 0.5 mM isopropyl-β-d-thiogalactopyranoside. Immediately after induction, cells were cooled to 20°C and harvested by centrifugation 18 hours after induction. The respective (His)_6_-tagged A-RRM1,2 protein was purified via nickel affinity chromatography on a Hi-trap column (GE Biosciences) followed by a Hi-trap Q column (GE Biosciences). The (His)_6_ purification tag was cleaved using the tobacco etch virus enzyme, and the cleavage mixture was then loaded onto Hi-Trap columns (GE Biosciences) to isolate the protein. Subsequently, A-RRM1,2 was loaded onto a HiLoad 16/600 Superdex 75 pg (GE Bioscience) gel filtration column and eluted into the desired buffer. Protein stock solutions were kept in a buffer consisting of 10 mM K_2_HPO_4_, 0.5 mM EDTA, 20 mM KCl, and 4 mM β-mercaptoethanol (BME; pH 6.5).

The UP1 protein (A1-RRM1,2) used in this study (residues 1 to 196) was subcloned into a pET28a vector and subsequently overexpressed as a C-terminal (His)_6_–tagged fusion protein in BL21 (DE3) cells and grown in LB broth. The C-terminal (His)_6_-UP1 was purified via nickel affinity chromatography on a Hi-Trap column (GE Bioscience) and subsequently loaded onto a HiLoad 16/600 Superdex 75 pg (GE Bioscience) gel filtration column and eluted into the desired buffer. Protein stock solutions were kept in a buffer consisting of 10 mM K_2_HPO_4_, 120 mM NaCl, 0.5 mM EDTA, and 5 mM dithiothreitol at pH 6.5. Protein homogeneity was confirmed by SDS-PAGE, and concentrations were determined using the theoretical molar extinction coefficient.

### NMR data acquisition

NMR spectra were recorded on a Bruker Avance (700 and 900 MHz) high-field spectrometers equipped with an HCN triple resonance cryoprobe and a *z*-axis field gradient accessory. Hydrogen-bonding was assigned by collecting exchangeable ^1^H imino spectra in 90% H_2_O, 10% D_2_O buffer containing 10 mM K_2_HPO_4_ (pH 6.5), 20 mM KCl, 0.5 mM EDTA, and 4 mM TCEP at 283 K using a Watergate NOESY (*t*_m_ = 200 and 250 ms) pulse sequence on the fully protonated RNA constructs. ^1^H-^1^H NOESY (*t*_m_ = 200) spectra were also collected in 100% D_2_O at 303 K on SLII^resist^ and the (DMA-135)-SLII^resist^ complex prepared with selectively deuterated rRTP ^2^H(3′,4′,5′5″) and fully deuterated pyrimidine ribonucleotide triphosphates (rYTPs). The samples were dissolved in 10 mM K_2_HPO_4_ (pH 6.5 before D_2_O exchange), 20 mM KCl, 0.5 mM EDTA, and 4 mM TCEP. All ^1^H-^13^C TROSY HSQC experiments were collected in the same buffer and at 303 K using RNA constructs prepared with selectively labeled ^13^C(rATP). All 2D NMR data were processed with nmrPipe/nmrDraw and analyzed using NMRFx analyst (*31*, *32*).

### MD simulation setup

The initial starting structure of SL2 wild type used for MD simulations was obtained from the Protein Data Bank (PDB code: 5 V16), and the SL2-Resist variant was generated using PyMOL’s mutation module (*33*). To accurately represent the resist variant, mutations were made within the bulge region of SL2 wild type (C132G and A133C) to generate the revertant mutation sequence to investigate the conformational profile. All initial structures were solvated within a cubic box, ionized, and simulated using the AMBER force field (ff3_leaprc.RNA.Shaw) at a temperature of 298 K (*34*). The Langevin thermostat maintained this temperature in the NVT ensemble. In addition, the simulation step size was set to 4 fs, with frames saved every 100 ps. The equilibration process was conducted at 298 K, which included 1000 steps of energy minimization. This was followed by a 1-ns simulation in an NVE ensemble (maintaining pressure at 1 atm using the Berendsen barostat), and a subsequent 2-ns simulation in an NPT ensemble. For long-distance electrostatic forces, the particle mesh Ewald method was used. To generate 10 distinct starting conformations for production runs, NPT simulations were carried out at 1 ns for each system. In this study, we performed 10 iterations of MD simulation using High Throughput Molecular Dynamics (HTMD). These trajectories were pivotal in constructing the MSM for each iteration and determining the starting positions for the subsequent iteration in line with the adaptive sampling algorithm. Adaptive sampling involved multiple rounds of simulations where trajectories from each round were analyzed to select initial coordinates for the next simulation round. The production simulations comprised 76 trajectories, each trajectory spanning 120 ns, and were distributed across 10 different epochs using the adaptive sampling protocols of HTMD.

### Markov state modeling

To elucidate the structures and substantial differences in bulge topology of clusters for SLII WT and SLII-resist, we used MSM for both systems using the PyEMMA2 package (*35*). For each RNA system, 10 MD simulations iterations yielded a total simulation time of approximately 8.6 ms. The MSM analysis was based on hydrogen bond distances and dihedrals between residues A134 and C138, U162 and U163 located in bulge of RNA structures. An important aspect of the MSM process is the TICA for reducing dimensionality and k-centers clustering (*36*–*38*). In addition, further analysis was conducted to evaluate the influence of the resistant mutations on the core hnRNP A1 binding motif (A136-G137) motif using Pymol.

### Isothermal titration calorimetry

RNA and protein samples used for calorimetry were prepared as described above. Calorimetric titrations were performed on a VP-ITC calorimeter (Microcal LLC) at 25°C using 10 mM K_2_HPO_4_, 20 mM KCl, 0.5 mM EDTA, and 4 mM BME (pH 6.5) buffer centrifuged and degassed under vacuum before use. The respective A1-RRM1,2 at 100 μM was titrated into ~1.4 ml of the respective RNA construct (SLII, SLII^CCC^, SLII^resist^) at 4 μM over a series of 32 injections set at 6 μl each. To minimize the accumulation of experimental error associated with batch-to-batch variation, titrations were performed in duplicate. Data were analyzed using the KinITC routines supplied with Affinimeter (*39*).

For the competition experiments, calorimetric titrations were performed on a VP-ITC calorimeter (Microcal LLC) at 25°C into 10 mM K_2_HPO_4_, 20 mM KCl, 0.5 mM EDTA, and 4 mM BME (pH 6.5) buffer centrifuged and degassed under vacuum before use. A-RRM1,2 at 100 μM was titrated into ~1.4 ml of 4 μM SLII:UP1 complex at a 1:1.2 molar ratio over a series of 32 injections set at 6 μl each. Likewise, UP1 at 100 μM was titrated into ~1.4 ml of 4 μM SLII:A-RRM1,2 complex at a 1:3.5 ratio over a series of 42 injections set at 6 μl each. In addition, this competition experiment was performed in the presence of DMA-135. UP1 at 100 μM was titrated into ~1.4 ml of 4 μM SLII:A-RRM1,2:DMA-135 at a 1:4:5 ratio, over a series of 42 injections set at 6 μl each. To minimize the accumulation of experimental error associated with batch-to-batch variation, titrations were performed in duplicate. Data were analyzed using KinITC routines supplied with Affinimeter (*39*).

### Cells and viruses

SF268 (human glioblastoma) cells were cultured at 37°C with 5% CO_2_ in RPMI 1640 medium supplemented with 10% fetal bovine serum (FBS, Gibco). Vero (African green monkey kidney) and RD (human embryonal rhabdomyosarcoma) cells were grown at 37°C with 5% CO_2_ in minimum essential medium (MEM) supplemented with 10% FBS. EV-A71 (TW/2231/98) was propagated in RD cells. EV-D68 (TW-02795-2014) was propagated in Vero cells.

### Isolation of DMA-135–resistant virus, 5′UTR cloning, and sequencing

SF268 cells were seeded in a six-well plate at a density of 3 × 10^5^ per well and cultured for 24 hours before virus infection. Cells were infected with undiluted EV-A71 stock (designated passage 0 virus) in the presence of 50 μM DMA-135 in RPMI 1640 supplemented with 2.5% FBS for 24 hours at 37°C. Medium was collected and saved (passage 1 virus). A 0.5-ml portion of medium was used to infect fresh SF268 cells again for 24 hours. Medium was harvested (passage 2). This process was repeated to passage 10. Virus titers of successive passages were determined by plaque assay using Vero cells (*26*). Passages 9 and 10 virus had titers comparable to passage 0 virus. Passage 9 virus was further characterized. Vero cells were infected with passage 9 virus and overlaid with 1% low-melting agarose (Invitrogen) in MEM and 2.5% FBS at 37°C for 4 days. Plaques were picked, virus was eluted, and virus titers were determined by plaque assay. One plaque-purified virus sample was selected for infection of SF268 cells for 24 hours. Total RNA was extracted from infected cells using a RNeasy kit (Qiagen). The 5′UTR region of EV-71A RNA was amplified using RT-PCR with primers EV1F: 5′-TTAAAACAGCCTGTGGGTTGC and EV745R: 5′-GTTTGATTGTGTTGAGGGTCA. The amplified cDNA fragment was cloned into plasmid pCRII-TOPO by TA cloning (Life Technologies). The sequence of the 5′UTR cDNA was determined using primer EV1F and compared to the sequence of the infectious clone used to prepare passage 0 virus.

### Construction of EV-A71 mutant virus with nt132 and nt133 mutations

The infectious clone of EV-A71, plasmid pEV71, and the dual luciferase reporter plasmid with nt 132 and nt 133 mutations, pRHF-EV71-5′UTR nts 132-133, were both digested with Apa I and Msc I. The ApaI-MscI fragment from pRHF-EV71-5′UTR nts 132-133 was purified and ligated into plasmid pEV71 digested with the same restriction enzymes. The resulting colonies were screened by sequencing to identify those harboring the two mutations. To prepare DNA for in vitro transcription, plasmid from a positive colony was purified and digested with EcoR I, fractionated in an agarose gel, and the infectious clone fragment purified. Full-length viral RNA containing the nt 132 and nt 133 mutations was synthesized by in vitro transcription using the infectious cDNA fragment. RNA was purified and transfected into SF268 cells. Supernatant was harvested 3 days after transfection. The mutant virus titer was determined by plaque formation with Vero cells.

To determine the effect of DMA-135 on mutant virus growth, SF268 cells were infected with wild-type or mutant virus at an MOI = 1 for 24 hours ± 50 μM DMA-135. Media were harvested, and virus titers were determined by plaque assays with Vero cells.

### Dual luciferase reporter assay

The mutant 5′UTR in pCRII-TOPO was amplified using primers EV1F and EV745R each containing a 5′ NotI restriction site. The PCR fragment was digested with NotI and cloned into the NotI site of pRHF to generate plasmid pRHF-EV71-5′UTR-nts 132-133 mut for luciferase assays (*40*). To assess the effects of putative suppressor mutations on IRES-dependent translation, capped and polyadenylated RNAs were prepared by in vitro transcription from the wild-type and mutant template DNAs linearized using AfeI (*26*). We refer to these RNAs as RLuc-(EV71/5′UTR)-FLuc for wild-type and RLuc-(EV71/5′UTR^revert^)-FLuc for the RNA containing the mutations in SLII. The RNAs were transfected into SF268 cells cultured without (i.e., vehicle) or with 50 μM DMA-135 for 2 days. Dual luciferase assays were then performed using the Dual Luciferase Assay kit (Promega) as described (*26*).

### Biotin-streptavidin pull-down assay of SLII-protein complexes

To assess the effects of putative suppressor mutations and DMA-135 on protein-SLII interactions, RNAs were prepared by in vitro transcription from wild-type and mutant SLII template DNAs in the presence of biotin-labeled uridine triphosphate. RNAs were capped and polyadenylated to maintain stability of the RNAs in cells. These RNAs were transfected into SF268 cells, and after 4 hours, various concentrations of DMA-135 were added to culture media. Twelve hours after transfection, cell lysates were prepared, and SLII-protein complexes were captured using streptavidin-sepharose. AUF1 and hnRNP A1 bound to SLII were detected by Western blot analyses (*26*). In some experiments, the SLII-associated proteins Ago2 and HuR were examined as well by Western blot. The primary antibodies used were as follows: anti-AUF1 rabbit polyclonal, 1:15,000 (Pocono Rabbit Farm & Lab, Canadensis, PA); anti-hnRNP A1 mouse monoclonal (Abcam), 1:200; anti-Ago2 rabbit polyclonal (Abcam), 1:200; and anti-HuR mouse monoclonal (Santa Cruz Biotechnology), 1:200.

### Knockdown of AUF1

Short hairpin (sh)–expressing plasmids that target AUF1 expression (shAUF1) or a shCTRL were transfected into SF268 cells, as described previously (*17*). AUF1 knockdown efficiency was monitored by Western blot from an aliquot of cells after 2 days in culture. In parallel, experiments were performed to assess the effects of combined AUF1 knockdown and DMA-135 treatment on, (i) virus titers for EV-A71–infected cells and (ii) SLII-protein complexes by protein-biotinylated SLII RNA pull-down assays.

### RIP and qRT-PCR analysis

As a control experiment, immunoprecipitation of endogenous protein-RNA complexes and qRT-PCR were used to examine the effects of DMA-135 on association of AUF1 with examples of its endogenous mRNA targets as previously described (*15*). The RNA samples analyzed in the present work were the same RNA samples used in our earlier study (*15*), except we measured the effects of DMA-135 on AUF1 association with cellular mRNAs *MYC*, *GFAT1*, and *rictor* (mTORC2 subunit); *GAPDH* mRNA served as a nonbound mRNA control (*27*). RNAs were analyzed by qRT-PCR using the following primer sets:

MYC (forward: 5′-acgaaactttgcccatagca-3′; reverse: 5′-gcaagg-agagcctttcagag-3′); GFAT1 (forward:5′-ccccagtcccacagaagtat-3′; reverse: 5′-aactgacagcattggctttg-3′); rictor (forward: 5′- ctaggtggca-ttgacattcagc-3′; reverse: 5′-ctaggaaacaaggaagcattcag-3′); and GAPDH (forward: 5′- gattgttgccatcaacgacc-3′; reverse: 5′-ccatggtg-gtgaagacacca-3′). Original samples were analyzed by Western blot to confirm specific immunoprecipitation of AUF1 [see supplementary figure 7 in (*15*)].
