# Supplementary material for: Enterovirus evolution reveals the mechanism of an RNA-targeted antiviral and determinants of viral replication
Source: Sci Adv. 2024 Feb 16;10(7):eadg3060. doi: 10.1126/sciadv.adg3060 (PMC10871541; doi:10.1126/sciadv.adg3060)
Supplement: Supplementary file 1 — Figs. S1 to S5 [file sciadv.adg3060_sm.pdf]

Science Advances
AAAS

Supplementary Materials for

Enterovirus evolution reveals the mechanism of an RNA-targeted antiviral and determinants of viral replication

Jesse Davila-Calderon *et al.*

Corresponding author: Blanton S. Tolbert, blanton.tolbert@pennmedicine.upenn.edu; Gary Brewer, brewerga@rwjms.rutgers.edu; Amanda E. Hargrove, amanda.hargrove@duke.edu

Sci. Adv. **10**, eadg3060 (2024)
DOI: 10.1126/sciadv.adg3060

This PDF file includes:

Figs. S1 to S5

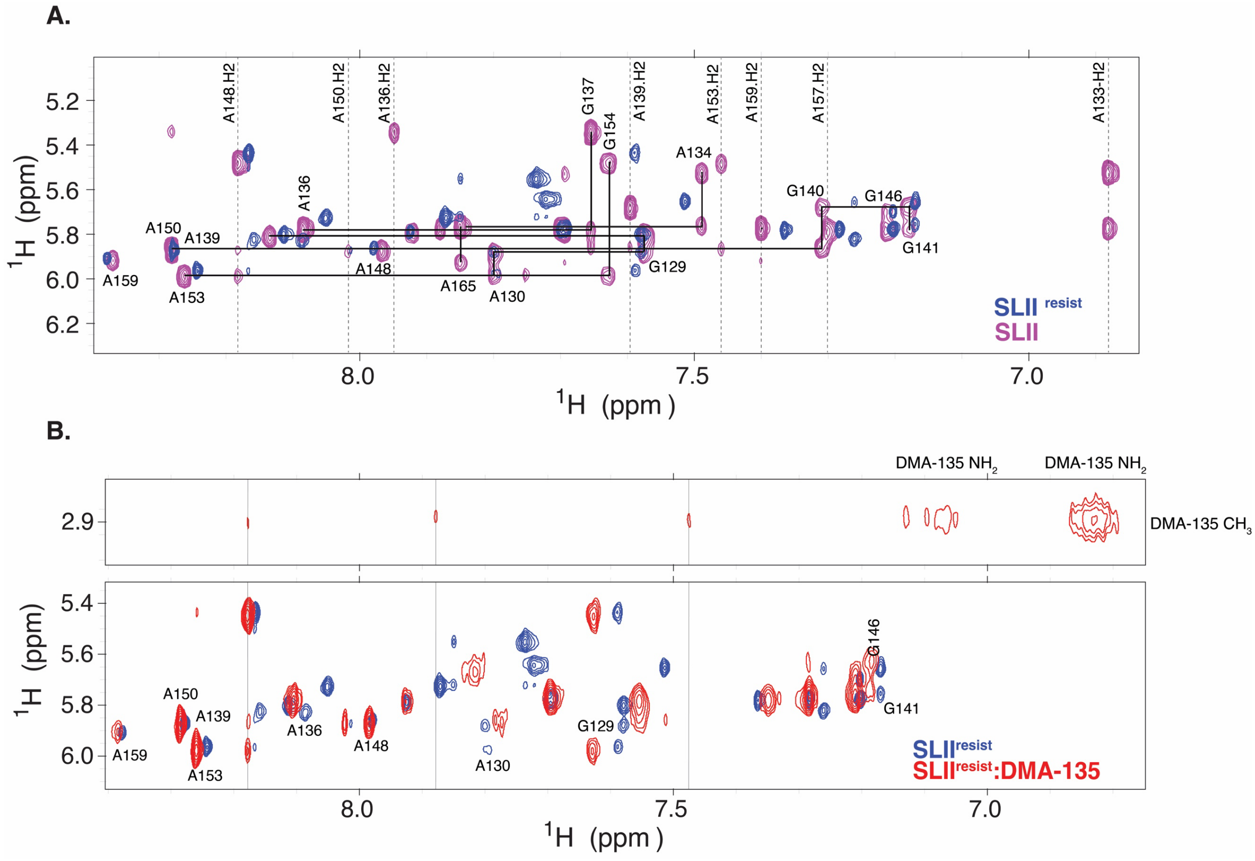

Figure S1. (**A**) Overlay of the ^1H-^1H NOESY spectra (t_m=200 ms) collected in of SLII (purple) and SLII^{resist} (blue) reveals that the C132G and A133C mutations abrogate base stacking within the bulge loop t while preserving the structure of the upper helix and apical loop. The black solid and dashed lines trace NOE spin systems of SLII. The spectra were recorded at 900 MHz in 10 mM K_2HPO_4 (pH 6.5 prior to exchanging in D_2O), 20 mM KCl, 0.5 mM EDTA and 4 mM BME D_2O buffer at 298 K. (**B**) Overlay of the ^1H-^1H NOESY spectra (t_m=200 ms) collected of free SLII^{resist} (blue) and the SLII^{resist}-(DMA-135) complex (red). The boxed region at 2.9 ppm indicates intermolecular NOEs between the methyl protons of DMA-135 and SLII^{resist}. Intramolecular NOEs between the methyl protons of DMA-135 and its amino protons are also observed. The spectra were recorded at 900 MHz in 10 mM K_2HPO_4 (pH 6.5 prior to exchanging in D_2O), 20 mM KCl, 0.5 mM EDTA and 4 mM BME D_2O buffer at 298 K.

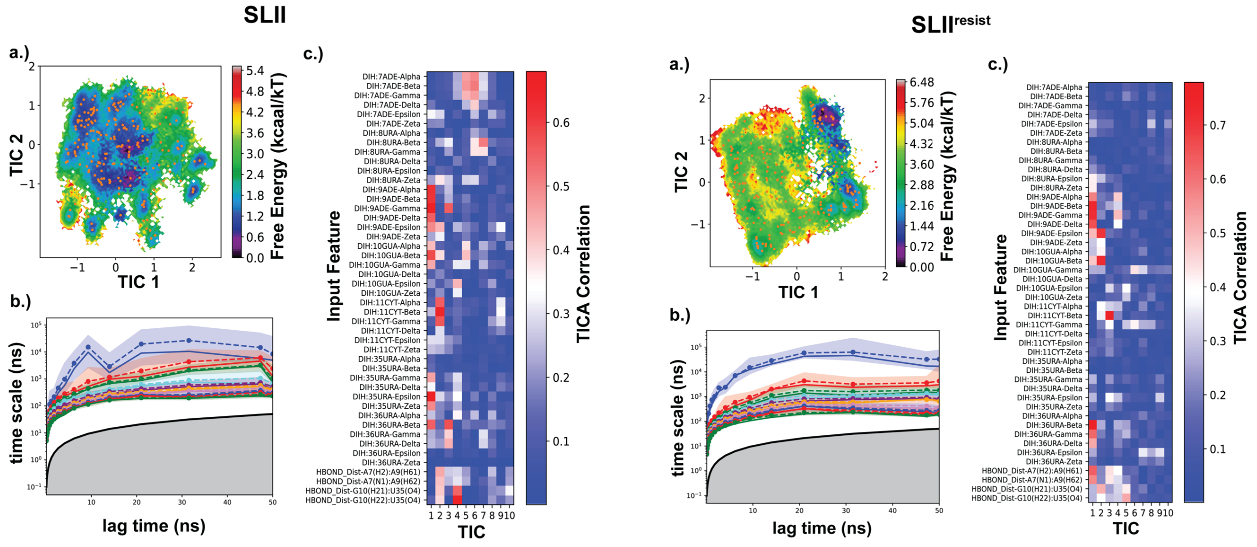

Figure S2. (a) free energy profiles of residues located in bulge structures for SLII (left) and SLII^{resist} (right). The free energy profiles are calculated using the MSM and are plotted as a function of the two slowest ICs from TICA. The locations of cluster centers within the TICA coordinate space are marked by orange dots. (b) This figure demonstrates the convergence of implied timescales for the ten slowest eigenvalues in the Markov State Model developed for residues in the bulge region SLII (left) and SLII^{resist} (right). The solid lines represent the average values of the implied timescales, while the shaded areas around these lines indicate the error margins. (c) The correlation coefficients of the independent components (ICs) derived from time independent component analysis (TICA) with the input features, such as dihedral angles of bulge residues and potential hydrogen bond donor-acceptor distances. The ICs represent the slowest-relaxing degrees of freedom in the system and are often interpreted as reaction coordinates. The correlation coefficients indicate the extent to which the ICs capture the variation in the input factors.

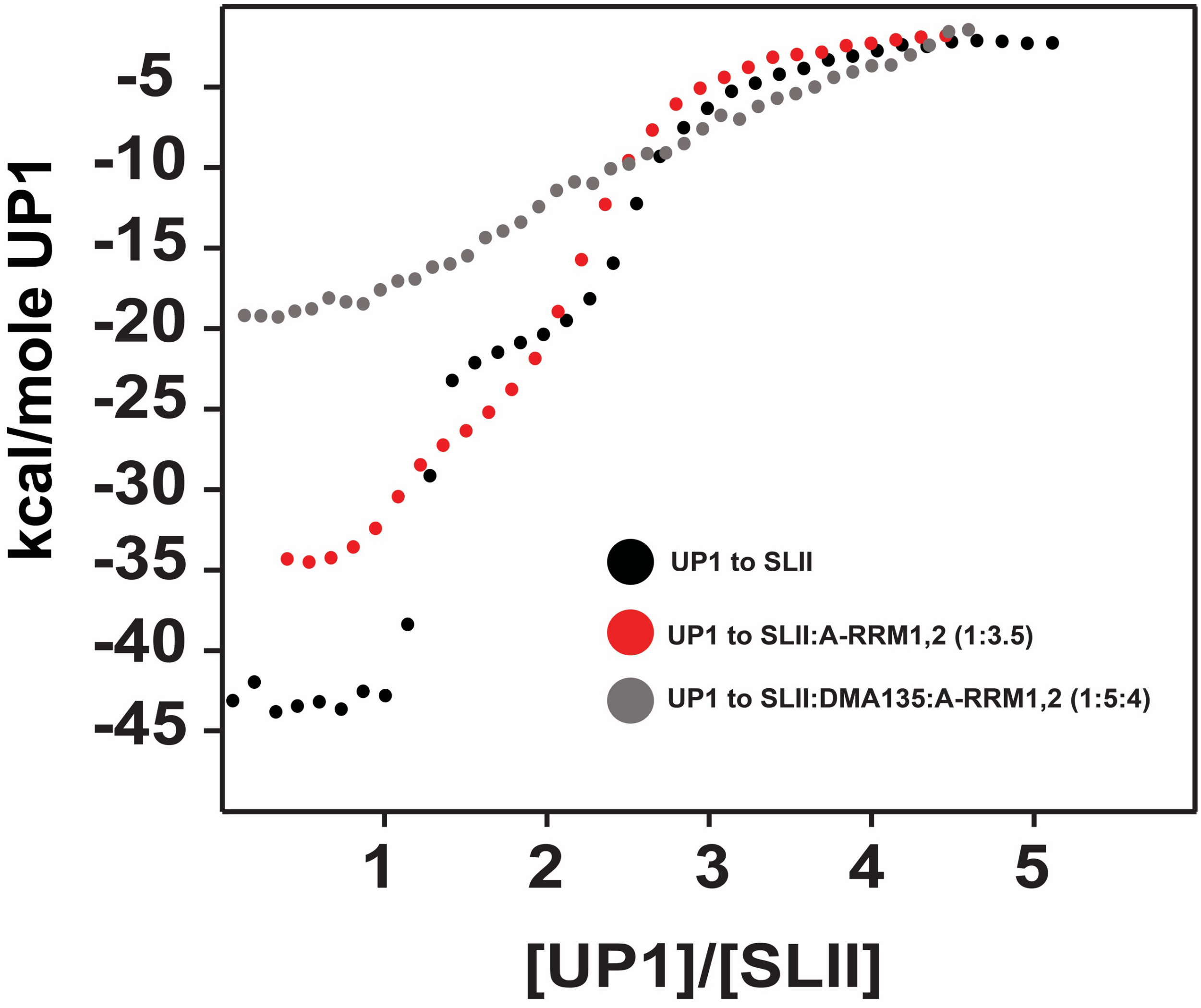

Figure S3. Comparison of the calorimetric titrations of UP1 into various complexes of SLII demonstrates competition for the bulge loop environment and the influence of DMA-135 to abrogate UP1 binding productively to the ternary complex. Titrations were performed in 10 mM K_2HPO_4 (pH 6.5), 20 mM KCl, 0.5 mM EDTA and 4 mM BME, and at 298K.

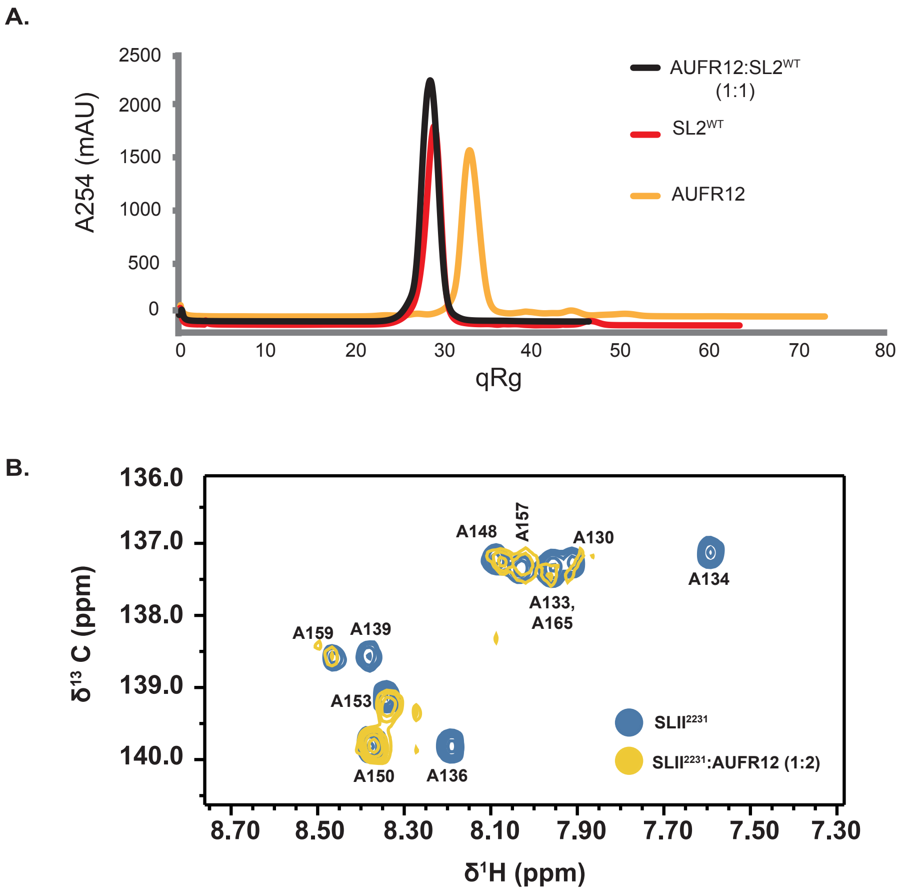

Figure S4 Biochemical evidence that A-RRM1,2 forms a stable and robust complex with SLII. (A) Analytical SEC titration of A-RRM1,2 into SLII. Titration was performed by pre-incubating 5 uM of SLII with equal molar amount of A-RRM1,2. The protein-RNA complex was resolved on a Superdex 200 10/300 GL column. (B) ^{1}H-^{13}C TROSY HSQC titration of A(^{13}C)-selectively labeled SLII with unlabeled A-RRM1,2. The blue correlation peaks correspond to free SLII and the yellow to the (A-RRM1,2)-SLII complex. The spectra were recorded at 900 MHz in 10 mM K_2HPO_4 (pH 6.5 prior to exchanging in D_2O), 20 mM KCl, 0.5 mM EDTA and 4 mM BME.

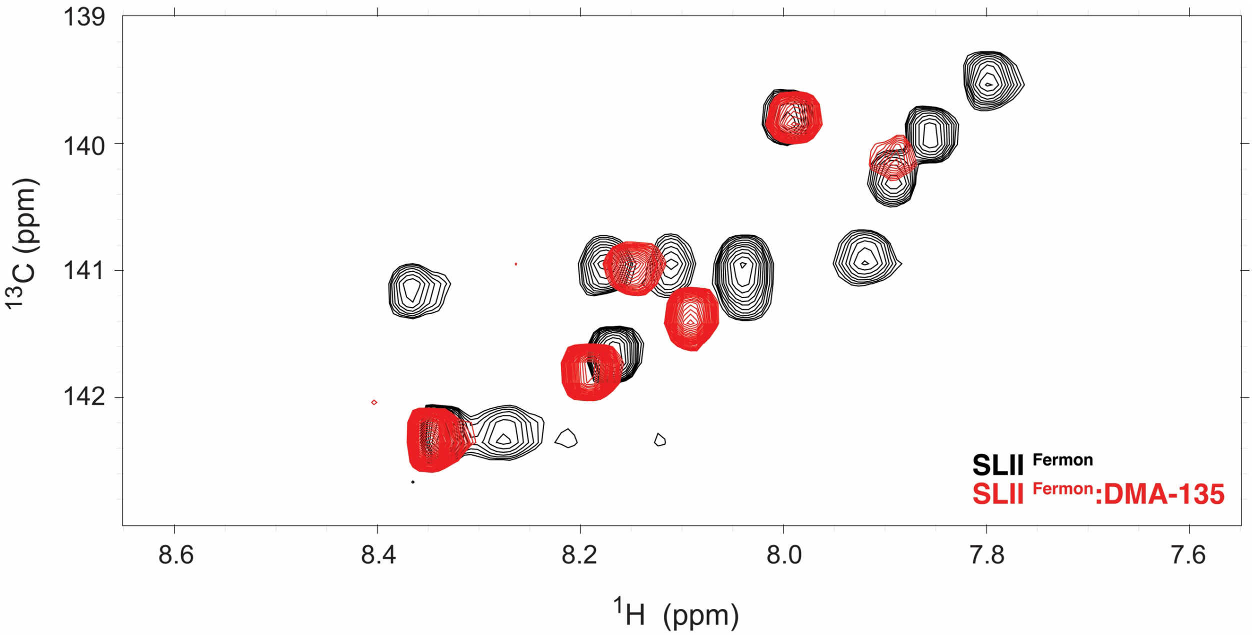

Figure S5. Single-point TROSY HSQC titration of A(^{13}C)-selectively labeled SLII^{Fermon} with DMA-135 at a 5-fold excess. The black correlation peaks correspond to free SLII^{Fermon} and the red to the SLII^{Fermon}-(DMA-135) complex. The spectra were collected at 900 MHz in 10 mM K_2HPO_4 (pH 6.5 prior to exchanging in D_2O), 20 mM KCl, 0.5 mM EDTA and 4 mM BME D_2O buffer at 298 K.
